# Common genetic variations in telomere length genes and lung cancer: a Mendelian randomisation study and its novel application in lung tumour transcriptome

**DOI:** 10.7554/eLife.83118

**Published:** 2023-04-20

**Authors:** Ricardo Cortez Cardoso Penha, Karl Smith-Byrne, Joshua R Atkins, Philip C Haycock, Siddhartha Kar, Veryan Codd, Nilesh J Samani, Christopher Nelson, Maja Milojevic, Aurélie AG Gabriel, Christopher Amos, Paul Brennan, Rayjean J Hung, Linda Kachuri, James D Mckay

**Affiliations:** 1 Genomic Epidemiology branch, International Agency for Research on Cancer/World Health Organization (IARC/WHO) Lyon France; 2 Cancer Epidemiology Unit, University of Oxford Oxford United Kingdom; 3 MRC Integrative Epidemiology Unit, Bristol Population Health Science Institute, Bristol Medical School (PHS) Bristol United Kingdom; 4 Department of Cardiovascular Sciences, University of Leicester Leicester United Kingdom; 5 NIHR Leicester Biomedical Research Centre, Glenfield Hospital Leicester United Kingdom; 6 Ludwig Lausanne Branch, Faculty of Biology and Medicine Lausanne Switzerland; 7 Institute for Clinical and Translational Research, Baylor College of Medicine Houston United States; 8 Lunenfeld-Tanenbaum Research Institute, Sinai Health Toronto Canada; 9 Departament of Epidemiology and Population Health, Stanford University Stanford United States; Translational Genomics Research Institute United States; McGill University Canada

**Keywords:** telomere length, lung cancer, GWAS, Mendelian randomisation, gene expression, Genome Stability, Human

## Abstract

**Background:**

Genome-wide association studies (GWASs) have identified genetic susceptibility variants for both leukocyte telomere length (LTL) and lung cancer susceptibility. Our study aims to explore the shared genetic basis between these traits and investigate their impact on somatic environment of lung tumours.

**Methods:**

We performed genetic correlation, Mendelian randomisation (MR), and colocalisation analyses using the largest available GWASs summary statistics of LTL (N=464,716) and lung cancer (N=29,239 cases and 56,450 controls). Principal components analysis based on RNA-sequencing data was used to summarise gene expression profile in lung adenocarcinoma cases from TCGA (N=343).

**Results:**

Although there was no genome-wide genetic correlation between LTL and lung cancer risk, longer LTL conferred an increased risk of lung cancer regardless of smoking status in the MR analyses, particularly for lung adenocarcinoma. Of the 144 LTL genetic instruments, 12 colocalised with lung adenocarcinoma risk and revealed novel susceptibility loci, including *MPHOSPH6*, *PRPF6*, and *POLI*. The polygenic risk score for LTL was associated with a specific gene expression profile (PC2) in lung adenocarcinoma tumours. The aspect of PC2 associated with longer LTL was also associated with being female, never smokers, and earlier tumour stages. PC2 was strongly associated with cell proliferation score and genomic features related to genome stability, including copy number changes and telomerase activity.

**Conclusions:**

This study identified an association between longer genetically predicted LTL and lung cancer and sheds light on the potential molecular mechanisms related to LTL in lung adenocarcinomas.

**Funding:**

Institut National du Cancer (GeniLuc2017-1-TABAC-03-CIRC-1-TABAC17‐022), INTEGRAL/NIH (5U19CA203654-03), CRUK (C18281/A29019), and Agence Nationale pour la Recherche (ANR-10-INBS-09).

## Introduction

Telomeres are a complex of repetitive TTAGGG sequences and nucleoproteins located at the end of chromosomes and have an essential role in sustaining cell proliferation and preserving genome integrity (de Lange, 2009). Telomere length progressively shortens with age in proliferative somatic cells due to incomplete telomeric regions replication (Watson, 1972) and low activity of the telomerase *TERT* in adult cells. The shortening of the telomere length results in cell cycle arrest, cellular senescence, and apoptosis in somatic cells (Harley et al., 1990). The maintenance of telomere length, which allows cancer cells to escape the telomere-mediated cell death pathways, is one feature related to the hallmarks of cancer (Hanahan and Weinberg, 2011).

Telomere length appears to vary between individuals and has been studied in relation to many diseases. In observational studies, telomere length is measured as the average length of telomeric sequences in a given tissue (Montpetit et al., 2014). Telomere length appears correlated across tissue types (Demanelis et al., 2020), and as such, leukocyte telomere length (LTL) is generally measured in epidemiologic studies as a proxy for telomere length in other tissues. Recently, LTL has been measured in 472,174 individuals from the UK Biobank (UKBB; Codd et al., 2021), and LTL was associated with multiple biomedical traits (i.e. pulmonary and cardiovascular diseases, haematological traits, lymphomas, kidney cancer, and other cancer types). Genetic analysis of LTL also revealed 138 genetic loci linked to LTL across a variety of different genes involved in telomere biology and DNA repair (Codd et al., 2021).

In the context of lung cancer, genetic variants at several loci have been associated with both LTL and lung cancer risk, including variants near the *TERT*, *TERC*, *OBFC1*, and *RTEL1* genes, fundamental to telomere length maintenance (McKay et al., 2008; Wang et al., 2008; Rafnar et al., 2009; Kachuri et al., 2016; McKay et al., 2017). The effects of the telomere-related variants appear more relevant to lung adenocarcinoma risk than other histologic subtypes (McKay et al., 2017; Landi et al., 2009). Accordingly, a causal relationship between LTL and susceptibility to lung cancer was observed using Mendelian randomisation (MR) approaches (Zhang et al., 2015; Haycock et al., 2017; Kachuri et al., 2019) as well as in observational studies that have associated directly measured telomere length with risk of lung cancer (Sanchez-Espiridion et al., 2014; Zhang et al., 2017).

The aim of the current work was to investigate the relationship between genetically predicted LTL and lung cancer, including lung cancer histological subtypes and smoking status. To this end, we conducted genome-wide correlations, MR, and colocalisation analyses to explore the relationship between LTL and lung cancer. We additionally undertook polygenic risk score (PRS) analysis using the LTL genetic instrument to explore the influence of LTL on the demographic, clinical, and molecular features of lung adenocarcinoma tumours.

## Materials and methods

### Reporting guidelines

The current study has been reported according to the STROBE-MR guidelines (Reporting Standards Document).

### Data

Genome-wide association studies (GWASs) summary statistics for lung cancer (29,239 cases and 56,450 controls) and stratified by histological subtype (squamous cell carcinoma, small-cell carcinoma, and adenocarcinoma) and smoking status (ever and never smokers) were obtained from the International Lung Cancer Consortium (ILCCO; McKay et al., 2017). All analyses of LTL requiring summary statistics used results from a GWAS of LTL in 464,716 individuals of European ancestry from the UKBB (Codd et al., 2021). Downstream analyses considered additional lung cancer risk factors, such as lung function and cigarette smoking. We obtained GWAS summary statistics for forced expiratory volume in 1 s (FEV_1_) and forced vital capacity (FVC) from a published UKBB analysis (Kachuri et al., 2020). For smoking behaviour traits, we used results from the GWAS and Sequencing Consortium of Alcohol and Nicotine use (GSCAN) consortium meta-analysis of cigarettes per day (continuous), smoking initiation (ever versus never), smoking cessation (successfully quit versus continuing), and age at smoking initiation (continuous; Liu et al., 2019) excluding the UKBB participants. For the obesity-related traits (continuous), we used the results from the UKBB and GIANT meta-analysis of BMI (Pulit et al., 2019) and waist-to-hip ratio (WHR; Pulit et al., 2019), or OpenGWAS data using UKBB participants (Elsworth et al., 2020) for high-density lipoprotein (HDL), triglycerides, and systolic and diastolic blood pressure. For the alcohol behaviour trait, we obtained the results from GSCAN phase 2 of drinks per week (continuous; Saunders et al., 2022). Colocalisation analyses of gene expression used lung tissue expression quantitative trait loci (eQTL) summary statistics from the Genotype-Tissue Expression (GTEx) data version 8.

Analyses of molecular phenotypes were performed using 343 lung adenocarcinoma samples of European ancestry from The Cancer Genome Atlas (TCGA) cohort with germline profile, RNA-sequencing, and epidemiological data available. Genotyping and imputation of germline variants have been described elsewhere (Gabriel et al., 2022). The total somatic mutation burden of TCGA samples was obtained from Ellrott et al., 2018, and DNA mutational signatures were extracted and attributed, as previously described (Gabriel et al., 2022). RNA-sequencing data were obtained from TCGA data portal using TCGA biolinks package in R (version 2.22.3; Colaprico et al., 2016). Telomere length measurement by whole-genome sequencing (WGS-measured TL, 655 samples across cancer sites) was retrieved from Barthel et al., 2017.

Tumour genomic characteristics were defined by the analyses of the TCGA data, including gene expression-based scores of telomerase activity (Barthel et al., 2017) and cellular proliferation (Thorsson et al., 2018), as well as the observed frequency of somatic homologous recombination-related events (represented as a homologous recombination repair deficiency score), and the average number of somatic copy number alteration within the tumours (Knijnenburg et al., 2018).

### Linkage disequilibrium score regression

Genetic correlations across traits were calculated using linkage disequilibrium score regression (LDSC) by the LDSC package (v1.0.0; Bulik-Sullivan et al., 2015). Linkage disequilibrium (LD) scores were generated on the 1000 Genomes Project Phase 3 reference panel with the HLA region excluded as provided by the package due to long range LD patterns. The genome-wide correlations that passed Bonferroni correction (adjusted p-values<0.05) were considered statically significant.

### Mendelian randomisation

MR is a method for interrogating relationships between putative risk factors and health outcomes by using genetic variants associated with the exposure of interest, typically obtained from GWAS, as instrumental variables. Assuming that fundamental MR assumptions are satisfied, this approach can be said to identify unbiased causal estimates. The genetic instrument for LTL was defined as the set of 144 genetic variants that were genome-wide significant (p<5x10^–08^) but not in linkage disequilibrium with each other (r^2^<0.01) and restricted to common genetic variation (minor allele frequency >1%) in European populations. Proxy variants in LD (r^2^>0.8, whenever possible) were chosen when a genetic variant was not available in the lung cancer GWAS. Primary MR analyses were conducted using the inverse-variance method with multiplicative random-effects (Yavorska and Burgess, 2017). Sensitivity analyses to horizontal pleiotropy and other violations of MR assumptions were performed using other MR estimation methods, such as weighted median, MR-Egger, contamination mixture model, MR-PRESSO, and MR-RAPS (Yavorska and Burgess, 2017; Sanderson et al., 2022). Multivariable MR (MVMR) methods included the inverse-variance weighted, MR-Egger, and least absolute shrinkage and selection operator (LASSO)-based methods (Yavorska and Burgess, 2017; Sanderson et al., 2019).

### Colocalisation methods

Unlike MR, where the goal is to assess the evidence for a causal effect of an exposure on an outcome, colocalisation is agnostic with respect to direction of effect and only assesses the probability that the two traits are affected by the same genetic variants at a given locus. Colocalisation can be viewed as a complementary approach for evaluating MR assumptions within specific genes or regions since strong evidence of colocalisation indicates overlap in genetic mechanisms affecting LTL and lung cancer. We used COLOC (v5.1.0; Wallace, 2020) to estimate the posterior probability for two traits sharing the same causal variant (PP_4_) in a 150 kb LD window, with PP_4_ >0.70 corresponding to strong evidence of colocalisation, as previously suggested (Wallace, 2020; Lopes et al., 2022). Priors chosen for the colocalisation analyses were p1=10^–3^, p2=10^–4^, and p12=10^–5^, or approximately, a 75% prior belief that a signal will only be observed in the LTL GWAS and less than 0.01% prior belief in favour of colocalisation between the two traits at a given locus (Giambartolomei et al., 2014). Conditioning and masking colocalisation methods were also used as they may identify putative shared causal variants in the presence of multiple causal variants present in a defined LD window (Wallace, 2021). We present the average PP_4_ from all methods as our posterior belief in favour of colocalisation between LTL and lung cancer risk. Multi-trait colocalisation based on a clustering algorithm was also performed using HyPrColoc (v1.0) to identify shared genetic signals with other lung cancer-related traits (Foley et al., 2021).

### Principal component analysis based on RNA-sequencing data

Read counts of RNA-sequencing data were normalised within (GC-content and gene length) and between (sequencing depth) lane procedures by EDASeq R package (version 2.28.0; Risso et al., 2011) and excluding low read counts. Principal component analysis was applied using singular value decomposition method, after excluding extreme outliers. Pathway analyses were conducted using Gene Set Enrichment Analysis software (GSEA, version 4.2.3; Subramanian et al., 2005) on gene annotations from Gene Ontology database. Pathway analyses were restricted to the top 500 genes positively and negatively correlated with each principal component that passed multiple-testing correction (Bonferroni-adjusted p-value<0.05 for 74,465 tests), which is the maximum number of genes supported by the online software.

The PRS for LTL was composed of the same 144 variants used in the MR analysis and was computed as the sum of the individual’s beta-weighted genotypes using PRSice-2 software (Choi and O’Reilly, 2019). Associations were estimated per SD increase in the PRS, which was normalised to have a mean of zero across lung adenocarcinoma samples of European ancestry within the TCGA cohort. The associations between the eigenvalues of the gene expression principal components (outcome) and demographic, clinical, and genomic features related to genome stability (predictors derived from TCGA published papers and TCGA data portal, except for the DNA mutational signatures [Gabriel et al., 2022]) were calculated using a multivariate linear regression model.

### Inferring PC2 gene expression signature based on RNA-sequencing data

The TCGA-lung adenocarcinoma (LUAD) tumour samples were split into training (70%, N=255) and validation (30%, N=108) datasets. Principal components analysis based on RNA-sequencing data was performed to summarise the gene expression profiles of lung adenocarcinoma tumours into five principal components in the training and validation datasets separately, as previously described (see ‘Principal component analysis based on RNA-sequencing data’ section in methods). Subsequently, we applied the partial least squares-based method called rigid transformation (Hubert and Branden, 2003) to align the first five principal components in both datasets. This method compares embeddings, low-dimensional representations in both datasets, performing a slightly rotation of principal components in order to translate and match them in both training and validation datasets. To select the most informative genes of PC2 in the training dataset, RNA levels of the genes correlated with PC2 (N=3914 out of 14,893 genes, FDR <0.05 for 14,893 tests) were selected as variables for the LASSO regression models. The LASSO tune parameters were chosen by resampling the training dataset (1000 bootstraps: root mean of SE=0.12 ± 0.0004, Lambda = 10 x 10^–10^, r^2^=0.99 ± 0.00007) using the tidymodels metapackage in R (v1.0.0; wrapper of glmnet). The 10 genes selected by the LASSO model were used to infer the gene expression signature of PC2 by adding up the scaled values of the log-normalised read counts of each gene multiplied by the respective LASSO regression coefficients. For validation purpose, the inferred PC2 signature was calculated in the validation dataset and compared with the observed principal components. After validation in the subset of the TCGA-LUAD cohort, the inferred PC2 signature was calculated in TCGA-LUSC dataset to compare differences between lung cancer histological subtypes.

## Results

### Genome-wide genetic correlations

**Figure 1.**
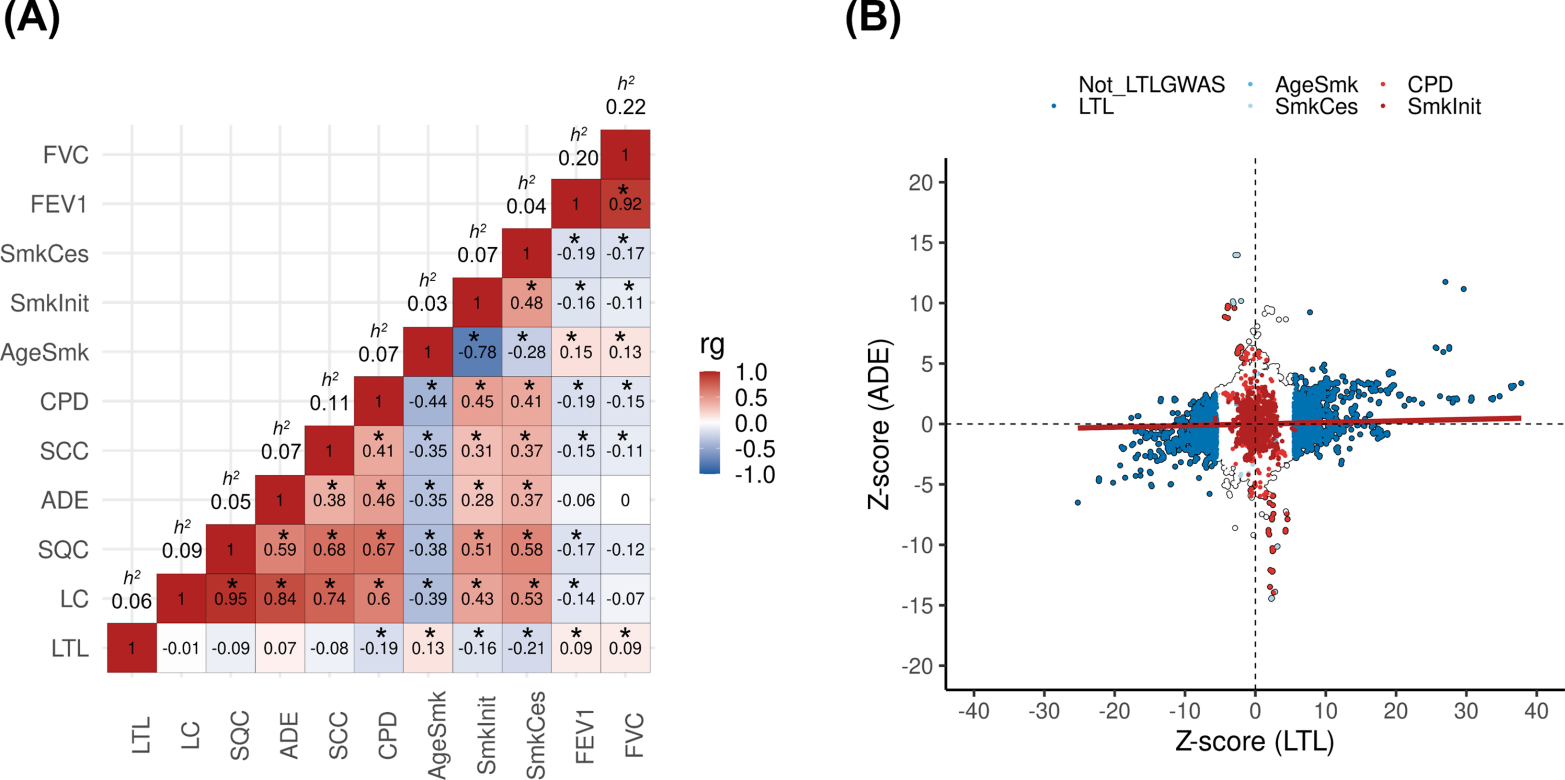
Genetic correlations between leukocyte telomere length (LTL) and lung cancer (LC) related traits. (**A**) Heatmap representing the genetic correlation analyses (rg) for LTL across LC, histological subtypes (lung adenocarcinoma [ADE], squamous cell carcinoma [SQC], and small-cell carcinoma [SCC]), smoking propensity (cigarettes per day [CPD], smoking cessation [SmkCes], Smoking initiation [SmkInit], and age of smoking initiation [AgeSmk]), and lung function related (forced vital capacity [FVC] and forced expiratory volume [FEV1]) traits. The black star indicates correlations that passed Bonferroni correction (p<4x10^–04^). Heritability (**h^2^**) as the proportion of the phenotypic variance caused by SNPs. (**B**) Plot of Z-scores (ADE versus LTL), restricting to the Hapmap SNPs (~1.2 million) but excluding HLA region. Genome-wide significant SNPs (p<5x10^–08^) for each trait were coloured (CPD in red, SmkInit in dark red, LTL in dark blue, AgeSmk in blue, SmkCes in lightblue, and not genome-wide hits for LTL or any other selected trait in white). Linear regression line was coloured in red.

The design of the study is represented in Figure 1—figure supplement 1. We first assessed the shared genetic basis of telomere length, lung cancer risk, and other putative lung cancer risk factors, such as smoking behaviours (age start smoking, smoking cessation, smoking initiation, and cigarettes per day) and lung function (FEV_1_ and FVC) using genome-wide correlations (Figure 1A). There was little evidence for genetic correlations by LDSC between LTL variants and lung cancer (r_g_ = −0.01, p=0.88) or when stratified by histologic subtypes (Figure 1A). Increasing LTL was genetically correlated with older age at smoking initiation (r_g_ = 0.13, p=3.0 × 10^–3^), and negatively correlated with smoking cessation: (r_g_ = −0.21, p=6.9 × 10^–09^), smoking initiation (r_g_ = −0.16, p=1.3 × 10^–10^), and cigarettes per day (r_g_ = −0.19, p=2.1 × 10^–08^). Longer LTL was genetically correlated with improved lung function, as indicated by increasing values of FEV_1_ (r_g_ = 0.09, p=5.1 × 10^–07^) and FVC (r_g_ = 0.09, p=1.1 × 10^–05^). To better understand the absence of genome-wide correlations between LTL and lung cancer, we visualised the Z-scores for each trait for approximately 1.2 million variants included in the LDSC analyses (Figure 1B). A subgroup of variants associated with longer LTL was correlated with increased lung adenocarcinoma risk, while the subgroup of smoking-behaviour associated variants, which also conferred an increased risk of lung adenocarcinoma, tended to have lower LTL.

**Figure 1—figure supplement 1.**
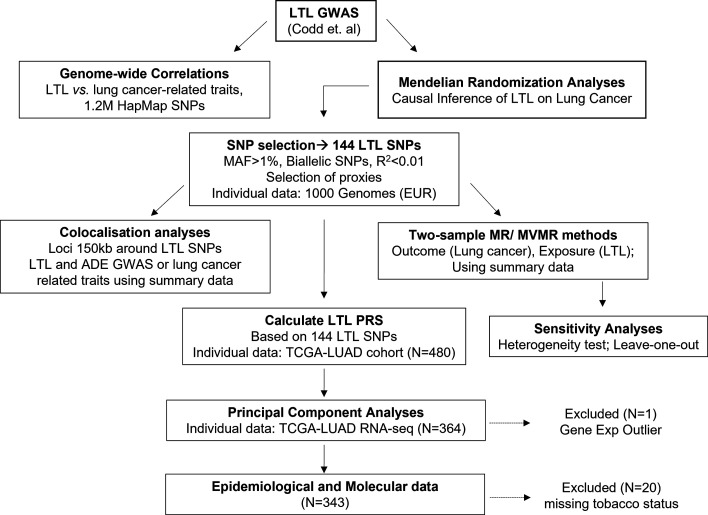
Design of the study. Upper: the leukocyte telomere length (LTL) variants were derived from the latest genome-wide association study (GWAS) in UK Biobank (UKBB) participants by Codd et al. Genome-wide correlations between LTL and lung cancer related traits were performed. Focus on a subset of LTL variants selected for Mendelian randomisation (MR) framework. Middle: selection of independent SNPs as LTL instrument for causal inference of LTL on lung cancer risk. Explore biological meaning of these variants using colocalisation methods and principal component analyses to summarise gene expression data. Bottom: calculate LTL polygenic risk score (PRS) based on the 144 SNPs and evaluate its association with principal components and epidemiological, and molecular data of lung adenocarcinoma tumours from The Cancer Genome Atlas (TCGA) dataset (TCGA-lung adenocarcinoma [LUAD]).

### MR analyses

From the 490 genetic instruments associated with LTL at genome-wide significance (p<5x10^–08^), 144 LTL genetic instruments, that explained ~3.5% of the variance in LTL, and were in low-linkage disequilibrium (r^2^<0.01) were used in MR analysis (Supplementary file 1a). As a sensitivity analysis, a PRS composed of these genetic instruments was associated with TL estimated from WGS in blood samples across TCGA cohorts (Beta = 0.03, 95%CI = 0.01–0.05, p=0.001) but was not associated with TL in tumour material from the same patients (Figure 2—figure supplement 1).

**Figure 2.**
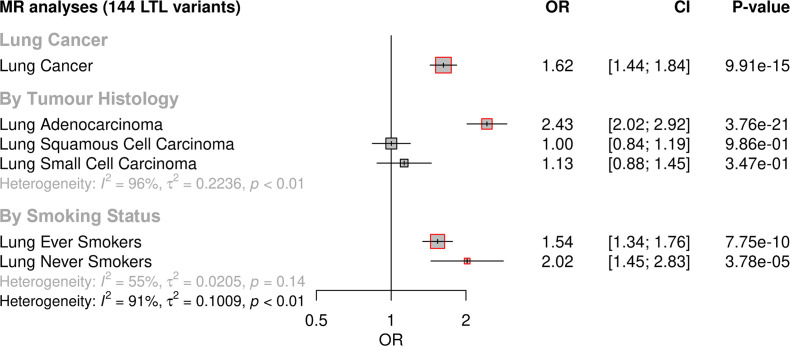
Genetically predicted leukocyte telomere length (LTL) association with lung cancer. Lung cancer (by histology or by smoking status) risk associations with the LTL instrument from the inverse-variance-weighted MR analyses are expressed as OR per SD increase in genetically predicted LTL. Statistically significant associations with p-values<0.05 (red square). Heterogeneity is estimated by the statistic I^2^, tau variance of subgroups (τ^2^), and p-values for Cochran’s Q heterogeneity measure.

MR analyses demonstrated that longer genetically predicted LTL was associated with increased lung cancer risk (OR = 1.62, 95% CI = 1.44–1.84, p=9.91 × 10^–15^) (Figure 2; Supplementary file 1). Longer LTL conferred the largest increase in risk for lung adenocarcinoma tumours (OR = 2.43, 95% CI = 2.02–2.92, p=3.76 × 10^–21^), but there was limited evidence of a causal relationship for other histologic subtypes, such as squamous cell carcinoma (OR = 1.00, 95% CI = 0.84–1.19, p=0.98) and small-cell carcinoma (OR = 1.13, 95% CI = 0.87–1.45, p=0.34; Figure 2, Supplementary file 1). However, our study was underpowered to detect an association between lung small-cell carcinoma and LTL at OR of 1.13 and considering alpha type-1 error rate of 5% (Figure 2—figure supplement 1). When stratifying the analyses by smoking status, LTL was associated with lung cancer risk in both never (OR = 2.02, 95% CI = 1.45–2.83, p=3.78 × 10^–05^) and ever smokers (OR = 1.54, 95% CI = 1.34–1.76, p=7.75 × 10^–10^; Figure 2, Supplementary file 1). Evidence for negative pleiotropy (supplementary file 1c) and heterogeneity (supplementary file 1d) was observed for all lung cancer outcomes except for squamous cell carcinoma. However, a significant association for LTL and lung cancer risk was found for methods robust to the significant directional pleiotropy (MR-Egger: lung cancer [OR = 2.35, p=3.37 × 10^–13^]; lung adenocarcinoma [OR = 4.48, p=7.30 × 10^–17^]; never smokers [OR = 6.84, p=2.07 × 10^–10^]; supplementary file 1b). Leave-one-out analyses detected only one outlier, rs7705526 in *TERT*, resulting in >10% change in MR effect size for associated lung cancer subtypes (supplementary file 1e). MVMR analyses considering instruments related to LTL and WHR, HDL, total triglycerides, systolic blood pressure, smoking, and alcohol intake, as well as multiple traits combined, suggested that the association between LTL and lung adenocarcinoma risk is independent of smoking propensity, obesity-related, and alcohol intake-related traits (supplementary file 1f, 1g).

**Figure 2—figure supplement 1.**
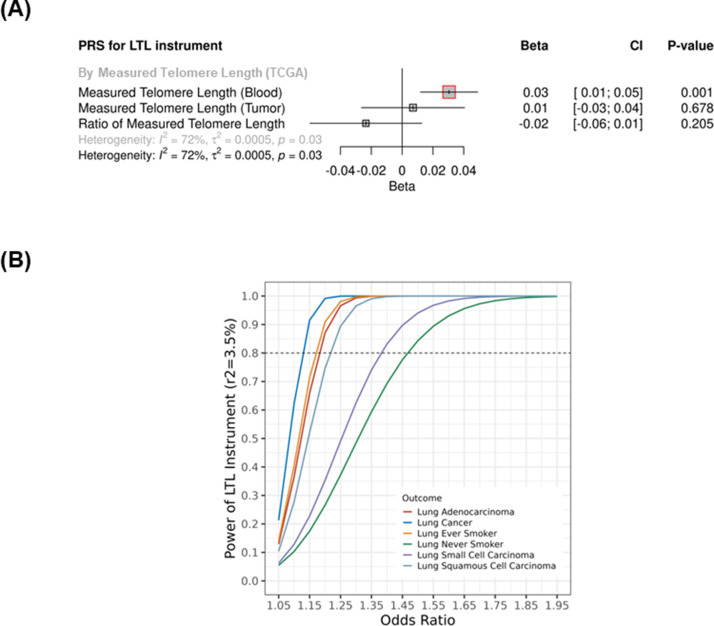
Sensitivity analysis of the genetically predicted leukocyte telomere length (LTL) Mendelian randomisation (MR) instrument. (**A**) Telomere length (TL) was measured by Barthel et al. in a subset of high-confident samples from The Cancer Genome Atlas (TCGA) cohorts using whole-genome sequencing (n=655). TL was directly measured in blood and tumour samples, and log(tTL/nTL) were also obtained from several TCGA cohorts. Associations were expressed as beta estimate per SD longer LTL in log scale. p-Values<0.05 (red square). Sex, age at diagnosis, cohort, and principal components of genetic ancestry (PC1-5) were used as covariates in the linear regression model. Heterogeneity is estimated by the statistic I^2^, tau variance of subgroups (τ^2^), and p-values for Cochran’s Q heterogeneity measure. (**B**) Power calculation by lung cancer strata considering a variance explained by the LTL instrument of 3.5% and alpha type-1 error rate of 5%.

### Colocalisation analyses

**Figure 3.**
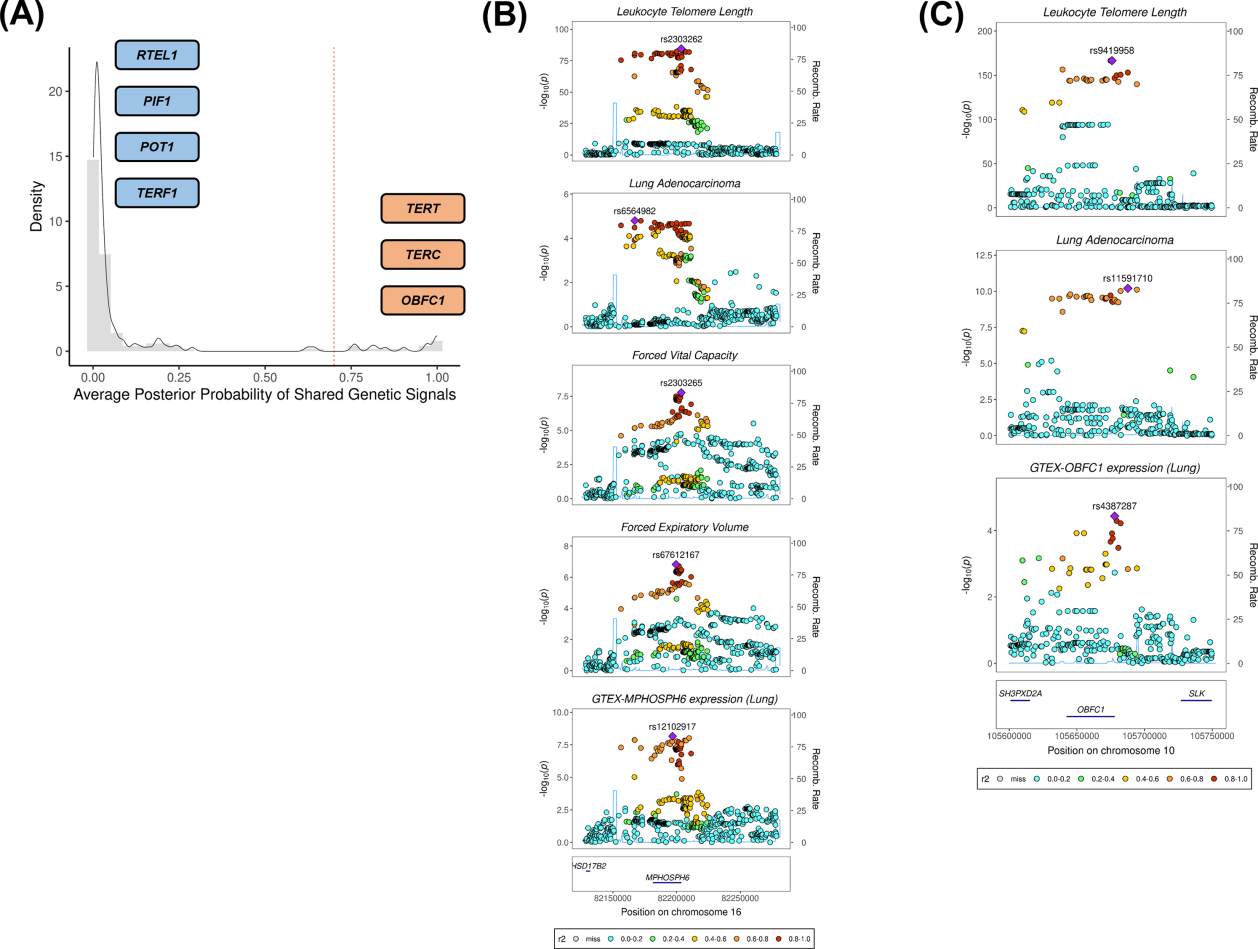
Colocalisation analyses for the genetic loci defined by the 144 leukocyte telomere length (LTL) variants. (**A**) Distribution of the average posterior probability for shared genetic loci between LTL and lung adenocarcinoma, highlighting in orange the telomere maintenance loci that colocalised (avg_PP4≥0.70) and in blue the ones where there was limited evidence for colocalisation (avg_PP4<0.70). Dashed red line represents the arbitrary avg_PP4 cutoff of 0.70. Representative stack plots for the multi-trait colocalisation results within (**B**) *MPHOSPH6* and (**C**) *OBFC1* loci, centred on a 150 kb LD window of rs2303262 and rs9419958 variants, respectively. Left Y-axis represents the –log10(p-values) of the association in the respective genome-wide association study for a given trait. The right Y-axis represents the recombination rate for the genetic loci. The X-axis represents the chromosome position. SNPs are coloured by the linkage disequilibrium correlation threshold (**r2**) with the query labelled SNP in European population. Sentinel SNPs within the defined LD window were labelled in each trait.

We investigated whether there was evidence of shared genetic signals between LTL and lung adenocarcinoma at loci centred on the 144 genetic instruments used in MR analyses using colocalisation methods (Figure 3A, supplementary file 1h). Loci with evidence of colocalisation between LTL and lung adenocarcinoma tended to be near genes that encode telomerase subunits and its associated complex, including genetic variants at *TERT* (5p15.33; rs116539972, rs7705526, rs61748181, rs71593392, and rs140648021), *TERC* (3q26.2; rs12638862 and rs146546514), and *OBFC1* (10q24.33; rs9419958 and rs139122544). Several colocalised loci mapped to genes that have not been previously linked to lung cancer risk at genome-wide significant level: *MPHOSPH6* (16q23.3; rs2303262), *PRPF6* (20q13.33; rs80150989), and *POLI* (18q21.2; rs2276182). Other telomere maintenance genes showed limited evidence of colocalisation with lung adenocarcinoma (i.e. *TERF1* and *PIF1*). For instance, while the *RTEL1* locus (20q13.33: rs117238689, rs115610405, rs35640778, and rs35902944) harboured variants associated with both LTL and lung adenocarcinoma (Figure 3—figure supplement 1), these signals appeared to be distinct and independent of each other (avg_PP3=0.999, avg_PP4=0.001; Figure 3A, supplementary file 1h).

**Figure 3—figure supplement 1.**
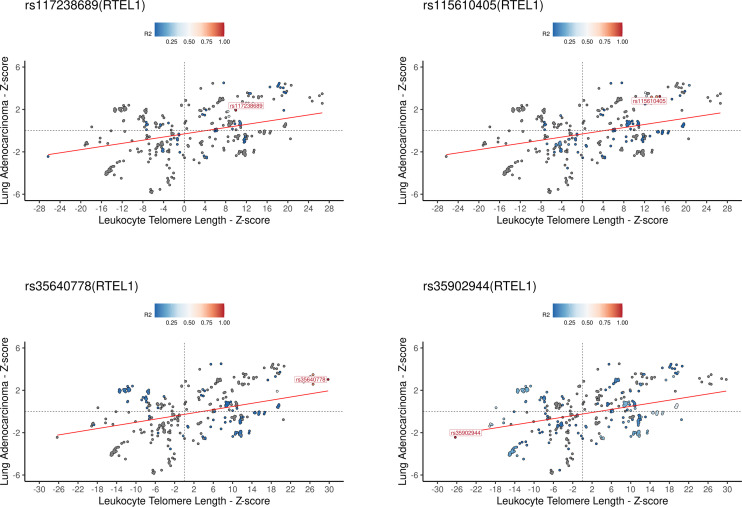
Association plots for leukocyte telomere length (LTL) and lung adenocarcinoma at *RTEL1* locus. Z-score plots for genetically predicted LTL and lung adenocarcinoma risk for the four LTL variants annotated for *RTEL1*. The genetic variants were coloured by the linkage disequilibrium correlation threshold (**r2**) with the query labelled SNP in a defined LD window of 150 kb centred on the query SNP in European populations. Z-score defined as the beta estimate divided by SE for each SNPs in the respective genome-wide association study.

We further evaluated whether the loci colocalised between LTL and lung adenocarcinoma also shared genetic signals with other traits related to lung cancer susceptibility (supplementary file 1i). Multi-trait analyses at the 16q23.3 locus colocalised rs2303262 with *MPHOSPH6* expression in lung tissue, FVC and FEV_1_, but not with any of the traits related to smoking behaviour (p=0.72; Figure 3B, supplementary file 1i). We additionally identified evidence of colocalisation (p=0.74) between lung adenocarcinoma, LTL, and gene expression in lung epithelial cells for two variants at the *OBFC1* locus: rs139122544 and rs9419958 (Figure 3C, supplementary file 1i).

### Genetically predicted LTL association with tumour features

**Figure 4.**
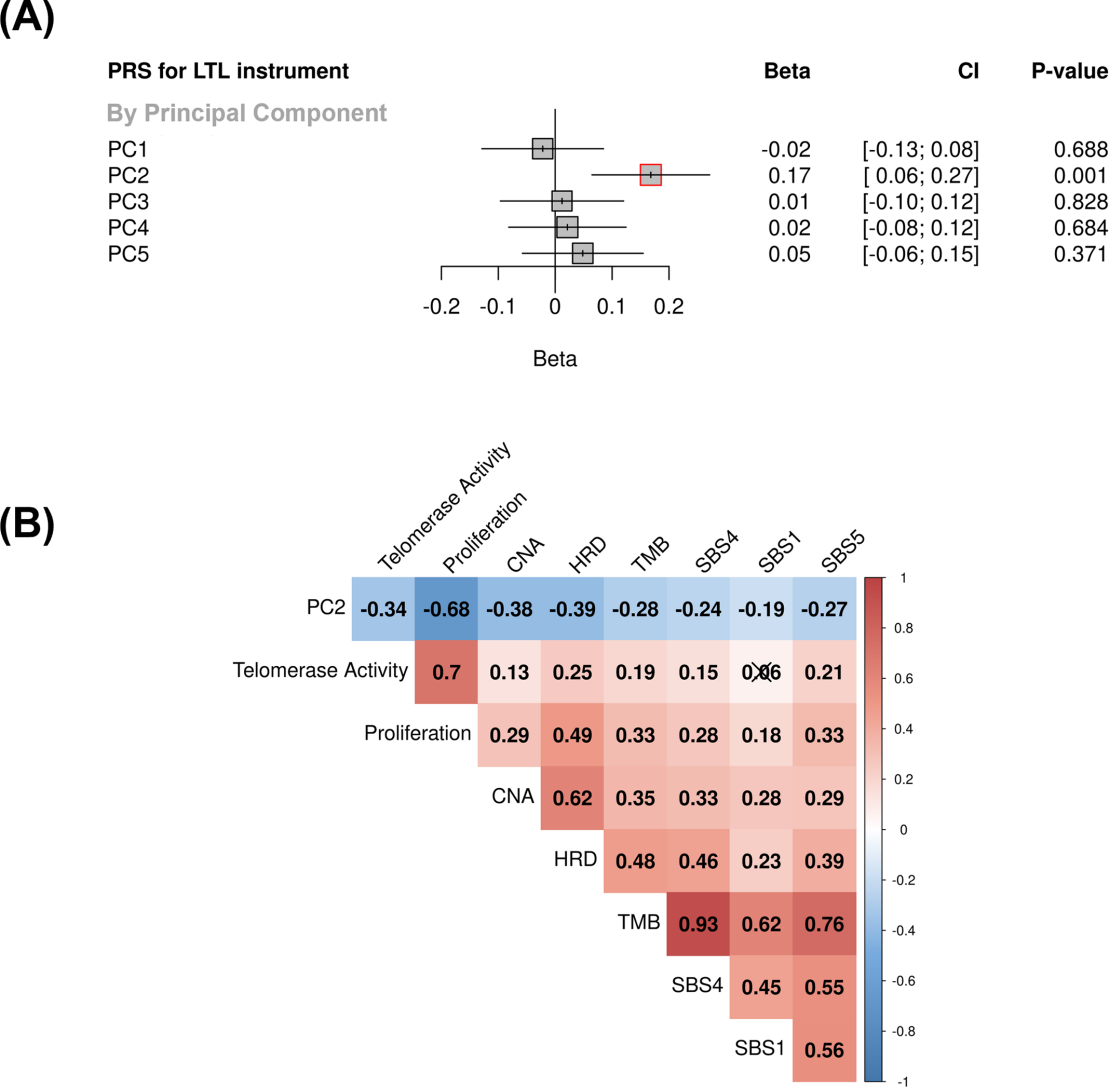
Associations between molecular expression patterns of lung adenocarcinoma tumours, LTL PRS, and The Cancer Genome Atlas (TCGA) features. (**A**) LTL PRS association with the first five principal components based on RNA-sequencing data of lung adenocarcinomas tumours (n=343). Results are expressed as beta estimate per SD increase in genetically predicted LTL. Linear regression model adjusted by sex, age, smoking status, and PC1-5 (genetic ancestry) covariates. Statistically significant associations with p-values<0.05 (red square). (**B**) Heatmap representing the correlations among PC2 and selected molecular features related to telomere length canonical roles. LTL = leukocyte telomere length; PRS = polygenic risk score; PC = principal component; TMB = tumour total mutation burden; HRD = homologous recombination deficiency, SBS (single base substitution DNA mutational signatures). SBS1 and SBS5 are DNA mutational signatures associated with age-related processes, and SBS4 is associated with tobacco smoking exposure. X-shaped marker to cross correlations with p-value>0.05.

We investigated the impact of genetically predicted LTL on lung adenocarcinoma tumour features by estimating molecular expression patterns within 343 lung adenocarcinomas tumours using principal component analysis in RNA-sequencing data. The first five components explained ~54% of the observed variance in the RNA-sequencing data (Figure 4, Figure 4—figure supplement 1). To explore the biological meaning of the five components, we performed pathway analyses for the top 500 genes with the highest loadings in each component (supplementary file 1j, supplementary file 1k). Overall, the genes correlated with each component tended to be enriched for specific cell signaling pathways (PC1: RNA processing; PC2: cell-cycle; PC3: metabolic processes; PC4: immune response; PC5: cellular response to stress and DNA damage; false discovery rate <5%; supplementary file 1l).

**Figure 4—figure supplement 1.**
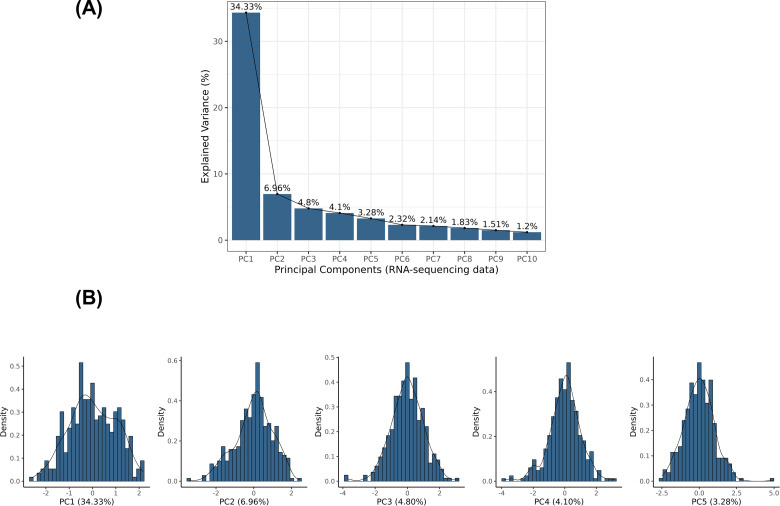
Principal component analysis (PCA) based on RNA-sequencing data. The RNA sequencing data from 343 primary lung adenocarcinoma tumour samples were retrieved. (**A**) Principal components analysis was applied to the centred log-transformed gene read counts, and the first five principal components were represented, which explained 53.5% of the variance in the gene expression for those samples. (**B**) The distributions of the first five principal components are represented in the density plots.

**Figure 5.**
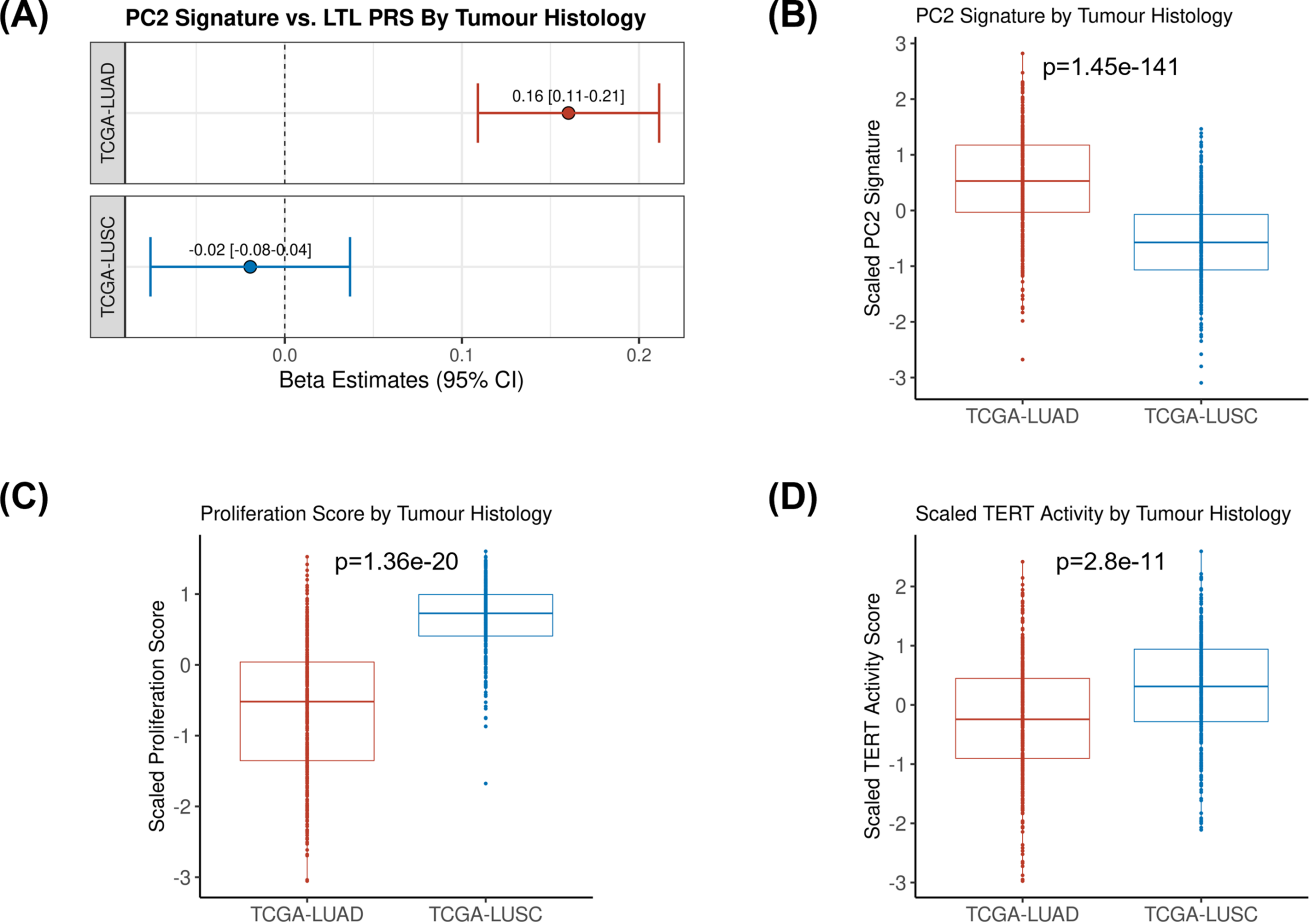
Comparing inferred PC2 gene expression signature by lung cancer histological subtypes. (**A**) Leukocyte telomere length (LTL) polygenic risk score (PRS) association with the 10-gene expression signature of PC2 in lung adenocarcinoma (The Cancer Genome Atlas [TCGA]-LUAD, N=343) and squamous cell carcinoma (TCGA-LUSC, N=338) cases from TCGA dataset. Results are expressed as beta estimate per SD increase in genetically predicted LTL. Linear regression model adjusted by sex, age, and PC1-5 (genetic ancestry) covariates, PC2 signature as outcome. Statistically significant associations with -values<0.05. Values per SD of (**B**) PC2, (**C**) proliferation score, and (**D**) telomerase/*TERT* activity gene expression signatures by lung cancer histological subtypes (TCGA-LUAD and TCGA-LUSC). p-Values derived from Student’s t-tests.

We then tested the association between the PRS composed of the 144 genetic instruments selected for MR analysis and the five components of gene expression within lung adenocarcinoma tumours (Figure 4A). The LTL PRS was positively associated with the second component (PC2) of tumour expression (Beta = 0.17, 95% CI = 0.12–0.19, p=1.0 × 10^–3^; Figure 4A). In multivariate analysis, higher values of PC2 tended to be associated with patients older at diagnosis (p=0.001), female (p=0.005), being never smokers (p=0.04), and diagnosed with early-stage tumours (p=0.002; Table 1). PC2 was also highly correlated with gene expression-based measure of cell proliferation and several genomic features related to genomic stability (Figure 4B). In multivariate analysis, higher values of PC2 were associated with reduced tumour proliferation (p=3.7 × 10^–30^), lower somatic copy number alternations (p=1.6 × 10^–05^), and higher tumour telomerase activity scores (p=1.6 × 10^–5^). Multivariate analysis also indicated that LTL PRS remained an independent predictor of PC2 when considering these genomic features (p=0.009; Table 1). It is noteworthy only nominal associations between LTL PRS and above-mentioned features and none remained statistically significant after correction for multiple testing (supplementary file 1m). We next inferred the gene expression signature of PC2, based on 10 genes informative of this component selected by the LASSO regression models, in both lung adenocarcinoma (TCGA-LUAD) and squamous cell carcinoma (TCGA-LUSC) cohorts (Figure 5, Figure 5—figure supplement 1). The association between LTL PRS and inferred PC2 was observed in TCGA-LUAD (p=0.001) but not in TCGA-LUSC (p=0.729) cases (Figure 5A). The inferred PC2 signature levels were higher in TCGA-LUAD than in TCGA-LUSC (Figure 5B), while higher proliferation rate (Figure 5C, p=1.45 × 10^–141^) and *TERT* activity (Figure 5D, p=1.36 × 10^–20^) were observed in TCGA-LUSC than in TCGA-LUAD cases. Of note, the low RNA levels of the telomere-related genes (less than five read counts), such as *TERT* and *TERC*, in both TCGA-LUAD and TCGA-LUSC tumour samples limited the direct comparison of these genes between these cohorts.

**Figure 5—figure supplement 1.**
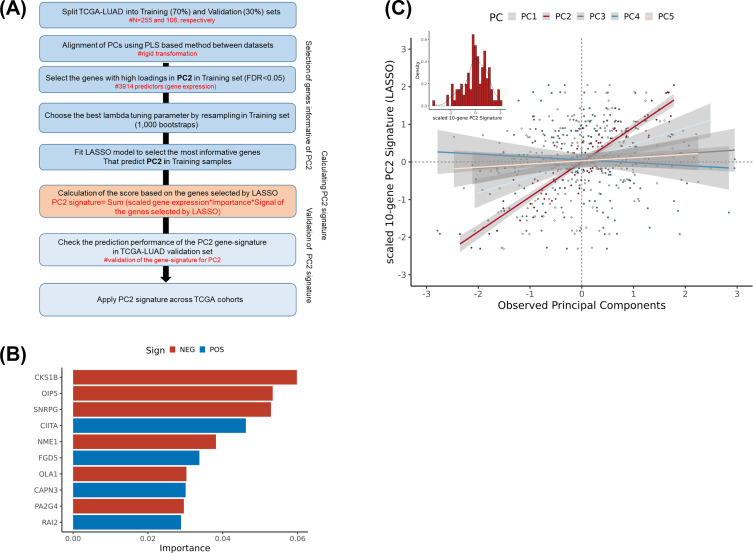
Generating inferred PC2 signature based on RNA-sequencing data. (**A**) Workflow for the generation of the PC2 signature. Calculate principal components based on RNA-sequencing data in both The Cancer Genome Atlas (TCGA)-lung adenocarcinoma (LUAD) training (N=255, 70%) and validation (N=108, 30%) datasets and use partial least square (PLS)-based method to align principal components (upper). Select the most informative genes correlated with the observed PC2 in the training dataset using least absolute shrinkage and selection operator (LASSO) regression model and validate it in the validation set (middle). Apply the PC2 signature to the TCGA-LUSC (lung squamous cell carcinoma) and TCGA-LUAD cohorts. (**B**) The ranked absolute coefficients/importance of the 10 genes selected by the LASSO models. Negative coefficients in red and positive ones in blue. (**C**) Scatter plot for the correlations between PC2 gene expression signature and observed principal components (PC1-5) based on RNA-sequencing data in the validation set. PC1 in light blue, PC2 in red, PC3 in grey, PC4 in blue, and PC5 in salmon colours.

**Table 1.** Association between PC2 (outcome) and lung adenocarcinoma tumour features in univariate and multivariate models (n=343).

Non-molecular features						
**Predictors**	**Univariate model**		**Multivariate model**			
	**OR/Beta (SE**)	**p-value**	**OR/Beta (SE**)	**p-value**		
Age at diagnosis^*^	0.17±0.05	0.001	0.17±0.05	0.001		
Gender (male)^†^	0.73±0.11	0.005	0.74±0.11	0.005		
Smoking status (ever)^†^	0.67±0.16	0.013	0.72±0.15	0.035		
Tumour stage (late)^†^	0.67±0.13	0.002	0.67±0.13	0.002		
**Molecular features**						
**Predictors**	**Univariate model**		**Multivariate model**			
	**Beta (SE**)	**p-value**	**Beta (SE**)	**p-value**		
LTL PRS ^‡^	0.17±0.05	0.001	0.10±0.04	0.009		
Telomerase activity	–0.37±0.05	9.34E-13	0.25±0.06	1.32E-05		
Proliferation	–0.69±0.04	3.30E-46	–0.80±0.06	3.66E-30		
Copy number alteration	–0.41±0.05	6.36E-16	–0.23±0.05	1.62E-05		
Homologous recombination deficiency	–0.4±0.05	8.32E-15	0.12±0.06	0.048		
Tumour total mutation burden	–0.28±0.05	1.37E-07	–0.09±0.24	0.695		
SBS1	–0.18±0.05	0.001	0.01±0.05	0.827		
SBS4	–0.24±0.05	6.36E-06	0.04±0.18	0.814		
SBS5	–0.27±0.05	4.84E-07	0.03±0.09	0.770		

SBS (single base substitution DNA mutational signatures); LTL = leukocyte telomere length; PC = principal component; PRS = polygenic risk score.

* age of diagnosis represented as beta estimate per 1 unit of SD.

† OR per 1 unit of SD.

‡ LTL PRS is adjusted by first five PC of genetic ancestry in the univariate model.

## Discussion

The maintenance of telomere length is one of the hallmarks of cancer, being critical for cell proliferation and genome integrity (Hanahan and Weinberg, 2011). Individual differences in telomere length, measured either directly or indirectly by germline determinates, have been linked with multiple diseases, including cancer susceptibility (Codd et al., 2021). The measurement of LTL within the UKBB has provided a resource for the development of a more powerful set of genetic instruments that capture a greater proportion of variation in LTL compared to previous studies (Codd et al., 2021). We applied genetic determinants of LTL to the largest GWAS of lung cancer to further characterise the role of telomere maintenance in lung cancer aetiology.

Using an MR analysis framework, we confirmed the previously reported relationship between genetically predicted longer LTL and increased risk of lung cancer. Our expanded genetic instrument detected systematic negative pleiotropy, which has not been observed in previous MR studies (Zhang et al., 2015; Haycock et al., 2017; Kachuri et al., 2019). Correcting for this pervasive directional bias resulted in substantially larger effects of LTL on risk of lung adenocarcinoma and lung cancer in never smokers, implying that LTL may even be more important to these phenotypes than previously estimated (Zhang et al., 2015; Haycock et al., 2017; Kachuri et al., 2019). Our observations in never smokers were also supported by multivariate MR analyses where adjustment for smoking did not attenuate the effect of LTL on lung cancer susceptibility. We used MVMR analysis (Sanderson et al., 2019) to assess the potential that factors, such as BMI, smoking, alcohol use, and other obesity related factors, may account for the association between LTL and lung cancer. While the influence of alternative unknown potential confounding factors cannot be excluded, the association of LTL with lung cancer risk was materially unaltered following adjustment for a range of potential confounders considered in our MVMR analyses.

Colocalisation analyses for the variants selected as the MR genetic instrument highlighted shared genetic signals between LTL and lung adenocarcinoma, including loci near genes related to telomere length maintenance (*TERT*, *TERC*, and *OBFC1*) and three genetic loci not previously linked to lung cancer susceptibility (*POLI*, *PRPF6*, and *MPHOSPH6*). The lung cancer risk allele of the *MPHOSPH6* sentinel variant (rs2303262) was associated with longer LTL, reduced pulmonary function, and increased *MPHOSPH6* gene expression in lung tissue. *MPHOSPH6* encodes an enzyme associated with the RNA exosome complex where it modulates RNA binding activity. *PRPF6* in 20q13.33 is involved in androgen binding and has been shown to promote colon tumour growth via preferential splicing of genes involved in proliferation (Adler et al., 2014). *POLI* is a member of the Y-family of DNA damage-tolerant polymerases involved in translesion synthesis (McIntyre, 2020). As part of its role in DNA repair and replication stress, *POLI* interacts with *TP53* to bypass barriers during DNA replication, which may confer a pro-survival effect to stem cells and cancer cells (Guo et al., 2021).

Despite the limited evidence of colocalisation between several loci important for telomere maintenance and lung cancer, the MR analyses restricting to the non-colocalised LTL SNPs pointed out that these loci might lie in the same causal pathway of LTL and lung cancer, highlighting the heterogeneity in the genetic effects of LTL loci.

We additionally identified the relationship between genetic determinants of LTL and a specific gene expression component in lung adenocarcinoma tumours. The aspect of this component associated with longer LTL was also associated with demographic and clinical features, such as never smoking, female, and early-stage tumours compared with other lung adenocarcinoma patients, which is an interesting but not completely understood lung cancer strata. This expression component also tended to be related to genomic features related to genomically stable tumours and strikingly associated with cell proliferation score, implying that this component might be a proxy for this feature. These results appear consistent with the canonical role of telomere length in preserving genome stability and cell proliferation (de Lange, 2009).

A plausible explanation for why long LTL was associated with an increased risk of lung cancer might be that individuals with longer telomeres have lower rates of telomere attrition compared to individuals with shorter telomeres. Given a very large population of histologically normal cells, even a very small difference in telomere attrition would change the probability that a given cell is able to escape the telomere-mediated cell death pathways (Aviv et al., 2017). Such inter-individual differences could suffice to explain the modest lung cancer risk observed in our MR analyses. However, it is not clear why longer TL would be more relevant to lung adenocarcinoma compared to other lung cancer subtypes. A suggestion may come from our observation that longer LTL is related to genomically stable lung tumours (such as lung adenocarcinomas in never smokers and tumours with lower proliferation rates) but not genomically unstable lung tumours (such as heavy smoking related, highly proliferating lung squamous carcinomas). One possible hypothesis is that histologic normal cells exposed to highly genotoxic compounds, such as tobacco smoking, might require an intrinsic activation of telomere length maintenance at early stages of carcinogenesis that would allow them to survive, and therefore, genetic differences in telomere length are less relevant in these cells. By contrast, in more genomically stable lung tumours, where TL attrition rate is more modest, the hypothesis related to differences in TL length may be more relevant and potentially explain the heterogeneity in genetic effects between lung tumours. Alternately, we also note that the cell of origin may also differ, with lung adenocarcinoma postulated to be mostly derived from alveolar type 2 cells, the squamous cell carcinoma is from bronchiolar epithelium cells (Sainz de Aja et al., 2021), possibly suggesting that LTL might be more relevant to the former.

One surprising finding from the genetic analysis was that despite the robust and large effects of LTL on lung cancer risk observed in the MR analyses, the genetic correlation between LTL and lung cancer was effectively null. The LDSC approach considers genetic variants across the entire genome, whereas the MR approach preferentially selects variants based on their association with LTL, restricting to those that achieved genome-wide significance. One possibility for the lack of genetic correlation between LTL and lung cancer is that genetic variants may differ in the direction that they influence these traits, and we used the smoking behaviour traits to exemplify that. For example, the subgroup of genetic variants noted at genome-wide significance from LTL studies was associated with increased LTL and lung cancer risk. However, the subgroup related to smoking behaviours which, in turn, are linked with increased lung cancer risk, tends to decrease LTL. If such opposing effects were widespread across the genome, it could account for the lack of genetic correlation between LTL and lung cancer estimated by LDSC and highlights the complex nature of the genetic variants that determine LTL and lung cancer risk.

Some limitations of this study should be acknowledged. A limited sample size might have limited the detection of an association between lung small-cell carcinoma and LTL. Our colocalisation approach is generally more conservative and may fail to accurately determine the posterior probability for shared genetic signals in the presence of multiple independent associations in a given locus (Hukku et al., 2021), which may be a reasonable explanation for the lack of colocalisation observed at *RTEL1* locus, and we stress that many of the variants that are COLOC negative are likely to be associated with lung cancer. Furthermore, the relatively small sample size of the lung adenocarcinoma cohort from TCGA may have reduced the power of our study, and larger cohorts of expression profiles tumours will be necessary to validate and explore some of our findings. The potential limitations such as collider bias within the lung adenocarcinoma case only study design should also be considered.

In conclusion, we describe an association between long genetically predicted LTL and lung cancer risk, which provides insights into how telomere length influences the genetic basis of lung cancer aetiology, including never smoking and female lung adenocarcinoma, which is an enigmatic subset of lung cancer. By using a novel framework to explore the biological implications of genetically complex traits, we unravel one gene expression component, highly correlated with proliferation rate score and other genomic stability-related features, associated with LTL in lung adenocarcinoma tumours. As far as we are aware, this is the first time an association between a PRS related to an aetiological factor, such as telomere length, and a particular expression component in the lung tumours is reported. Our findings suggest that lung adenocarcinoma patients with longer LTL might have more genomically stable tumours than the ones with shorter LTL, shedding some light on telomere biology in those tumours.

## Data Availability

Lung cancer GWAS summary statistics obtained from ILCCO can be accessed by the database of Genotypes and Phenotypes (dbGAP) under accession phs000876.v1.p1. The GWAS summary statistics for tobacco-smoking behaviors (GSCAN: https://conservancy.umn.edu/handle/11299/201564), LTL (https://figshare.com/s/caa99dc0f76d62990195), and GTEx version 8 (downloaded via GTEx google cloud resource) are publicly available. Germline data of TCGA cohorts were accessed by dbGAP under accession number phs000178.v11.p8 and project application #2731. RNA-sequencing data from TCGA cohorts were retrieved from GDC open-access data portal (https://portal.gdc.cancer.gov/) using TCGAbiolinks package in R. TCGA-related data are publicly available as described in the data section. The code for LDSC analysis is available at: https://github.com/bulik/ldsc/wiki/Heritability-andGenetic-Correlation. The codes used in this study for two-sample MR, colocalisation, multi-trait colocalisation, and principal component analyses are available at https://github.com/ricardocortezcardoso/Telomere_Length_Code, (Penha, 2023a, copy archived at swh:1:rev:f365df300919c46bb99a96b4040d90576fc878e2). Plots were created using R packages ‘meta’ (v5.5, forest plots), ‘corrplot’ (v0.92, correlation matrix), and ‘ggplot2’ (v3.3.6). The R package to generate stackplots for visualisation of the multi-trait colocalisation results is available at https://github.com/jrs95/gassocplot, (Penha, 2023b, copy archived at swh:1:rev:ae6a59dff2e43d39eead3d483af1d50f151c3d5b).

## Additional files

Supplementary file 1. Supplementary tables (a-m). (a) SNPs selected as leukocyte telomere length (LTL) instrument for the Mendelian randomisation (MR) analyses. (b) MR analyses across methods for the 144 LTL SNPs. (c) Test for directional pleiotropy using MR Egger for the 144 LTL SNPs. (d) Heterogeneity tests for the 144 LTL SNPs. (e) Leave-one out analyses for the 144 LTL SNPs. (f) Multivariable MR analyses. (g) Multivariable MR analyses for LTL and lung cancer adjusting for obesity- and alcohol-related traits. (h) Summary of the colocalisation results for the LTL instrument based on 144 variants. (i) Summary of the multi-trait colocalisation results for the 12 colocalised genetic loci. (j) Top 500 genes positively correlated with the first five principal components. (k) Top 500 genes negatively correlated with the first five principal components. (l) Pathway analysis on the top 500 genes positively and negatively correlated with the first five principal components based on RNA-seq data of 343 ADE cases from The Cancer Genome Atlas (TCGA) dataset using Gene Set Enrichment Analysis (GSEA). (m) Association of LTL polygenic risk score (PRS) with the clinical and molecular features of lung adenocarcinoma tumours.

MDAR checklist

## Appendix 1

### Additional methods: Datasets

#### UK Biobank

The UK Biobank (UKBB) is a large cohort with more than 500,000 individuals from the United Kingdom who were recruited between 2006 and 2010, as previously described (Bycroft et al., 2018). At the time of recruitment, individuals provided electronic signed consent, answered questions regarding socio-demographic, lifestyle, health-related factors, and physical measures. The genetic data were obtained using the UK BiLEVE and the UKBB Axiom arrays. UKBB has ethical approval from the National Information Governance Board for Health and Social Care and the North-West Multicentre Research Ethics Committee (Ref: 11/NW/0382).

The latest genome-wide association study (GWAS) summary statistics of leukocyte telomere length (LTL; https://figshare.com/s/caa99dc0f76d62990195) were performed on 464,716 UKBB participants of European ancestry with 19.4 million imputed variants ( minor allele frequency≥ 1% and imputation quality score ≥0.3), adjusting by age, sex, array, and the first 10 principal components. LTL was measured from UKBB baseline samples, and values were log-transformed and Z-standardised.

The forced expiratory volume in 1 s (FEV_1_) and forced vital capacity (FVC) GWAS summary statistics were conducted on 372,750 and 370,638 individuals of European genetic ancestry, respectively, who had spirometry data and sample passed quality control of genetic data (Hardy–Weinberg equilibrium at p>1 × 10^−5^, MAF >0.5%, and imputation quality score ≥0.3). Linear regression models were applied to standardised Z-scores for FEV_1_ and FVC, adjusting by age, sex, genotyping array, and 15 principal components. These GWAS summary statistics are available by the corresponding authors of the original article upon reasonable request (see Kachuri et al., 2020).

#### International lung cancer consortium

The International Lung Cancer Consortium (ILCCO) is an international group of lung cancer researchers for sharing comparable data from ongoing lung cancer case-control and cohort studies. The lung cancer GWAS summary statistics used in the current work is derived from a collaborative effort between Transdisciplinary Research of Cancer in Lung of the ILCCO (TRICL-ILCCO) and the Lung Cancer Cohort Consortium (LC3). All participants in these studies signed an informed consent, approved by the local internal review board or ethics committee.

The lung cancer GWAS summary statistic (29,266 cases and 56,450 controls of European descent; McKay et al., 2017) is meta-analysis of the combined imputed genotypes from OncoArray series (14,803 cases and 12,262 controls) and previous lung cancer GWAS (IARC, MDACC, SLRI, ICR, Harvard, NCI, Germany, and deCODE studies; 44,188 controls and 14,436 cases). The meta-analysis only considered imputed variants with MAF >1%, imputation quality score ≥0.3, and with little evidence for heterogeneity in effect between the studies (Cochran’s Q statistic: p>0.05), adjusting by sex, age, and principal components. Additionally, analyses were conducted by lung cancer histological subtype (adenocarcinoma [11,273 cases and 55,483 controls], squamous cell carcinoma [7426 cases and 55,627 controls], and small-cell carcinoma [2664 cases and 21,444 controls]). Of note, histological subtype information was not available for all studies.

#### The GWAS and Sequencing Consortium of Alcohol and Nicotine use consortium

The GWAS and Sequencing Consortium of Alcohol and Nicotine use dataset is derived from an international genetic association meta-analysis consortium for tobacco smoking and alcohol consumption traits across 30 studies and up to 1.2 million individuals (Liu et al., 2019). For the GWAS summary statistics used in the current study, UKBB participants were excluded from the meta-analysis, and only genetic variants present in more than 19 studies, MAF >1%, and imputation quality score >0.3 were considered. The smoking traits were defined as follow: cigarettes per day (CPD; N=337,334; continuous variable defined as average number of cigarettes smoked per day, either as a current smoker or former smoker), smoking initiation (SmkInit; N=1,232,091; categorical variable defined as ever smokers versus never smokers), smoking cessation (SmkCes; N=547,219; categorical variable defined as current smokers versus former smokers), and age of initiation (AgeSmk; N=341,427; continuous variable defined as the age at which an individual first became a regular smoker). Age, sex, and genetic principal components were used as covariates in all analyses. GWAS summary statistics were downloaded from https://conservancy.umn.edu/handle/11299/201564.

The Genotype-Tissue Expression (GTEx) project data is a project that aims to investigate the effects of genetic variants in transcriptome and their impact on regulatory mechanisms of a traits and diseases (GTEx Consortium, 2020). The GTEx data contain 15,201 RNA-sequencing samples from 49 tissues of 838 postmortem donors. For the current study, we accessed version 8 of the GTEx program to obtain the effect estimates and risk alleles of eQTL genetic variants (in normal lung tissue) around the queried SNPs for the multi-trait colocalisation analyses.

### The Cancer Genome Atlas

The Cancer Genome Atlas (TCGA) is currently the biggest resource of cancer genomics, with more than 20,000 primary cancer and matched normal samples molecularly characterised across 33 cancer types with available demographic and clinical data.

For the current study, the access for the germline data of TCGA dataset was obtained via TCGA project #2731 via dbGAP (phs000178.v11.p8). Genotyping and imputation from TCGA data were performed in-house and described elsewhere (Gabriel et al., 2022). Briefly, germline variants with low genotyping call rate (<97%), MAF <1%, strong deviation (p-value<10^–8^) from the Hardy–Weinberg equilibrium, and allele frequency differing more than 20% from 1000 Genomes (1000 G) within European ancestry group were removed. Phasing was performed using Eagle (v2.4.1; Gabriel and Lipinski, 2021) with the 1000 genomes phase 3 reference panel, and Minimac4 (v1.0.1; Gabriel and Lipinski, 2021) was used to perform the imputation (window size of 500 kb; Das et al., 2016). Samples with discrepancies between genetic inferred and self-reported sex as well as with high inter-sample relatedness (PI_HAT >0.185%) were removed.

Of the 480 lung adenocarcinoma cases with genotype data, 364 cases had available tumour RNA-sequencing data (more details in Data availability section). We excluded 20 cases due to missing information for smoking status (ever smokers versus never smokers) from the downstream analyses. After summarising the normalised log-transformed read counts for all genes in two dimensions, one sample was removed due to extreme gene expression profile in comparison with all the lung adenocarcinoma tumours. Thus, 343 lung adenocarcinoma tumour samples were selected for the principal component analysis and association with LTL polygenic risk score (PRS), molecular, clinic, and demographic variables of TCGA dataset.

## References

[bib1] Adler AS, McCleland ML, Yee S, Yaylaoglu M, Hussain S, Cosino E, Quinones G, Modrusan Z, Seshagiri S, Torres E, Chopra VS, Haley B, Zhang Z, Blackwood EM, Singh M, Junttila M, Stephan JP, Liu J, Pau G, Fearon ER, Jiang Z, Firestein R (2014). An integrative analysis of colon cancer identifies an essential function for prpf6 in tumor growth. Genes & Development.

[bib2] Aviv A, Anderson JJ, Shay JW (2017). Mutations, cancer and the telomere length paradox. Trends in Cancer.

[bib3] Barthel FP, Wei W, Tang M, Martinez-Ledesma E, Hu X, Amin SB, Akdemir KC, Seth S, Song X, Wang Q, Lichtenberg T, Hu J, Zhang J, Zheng S, Verhaak RGW (2017). Systematic analysis of telomere length and somatic alterations in 31 cancer types. Nature Genetics.

[bib4] Bulik-Sullivan BK, Loh PR, Finucane HK, Ripke S, Yang J (2015). LD Score regression distinguishes confounding from polygenicity in genome-wide association studies. Nature Genetics.

[bib5] Bycroft C, Freeman C, Petkova D, Band G, Elliott LT, Sharp K, Motyer A, Vukcevic D, Delaneau O, O’Connell J, Cortes A, Welsh S, Young A, Effingham M, McVean G, Leslie S, Allen N, Donnelly P, Marchini J (2018). The UK Biobank resource with deep phenotyping and genomic data. Nature.

[bib6] Choi SW, O’Reilly PF (2019). PRSice-2: polygenic risk score software for biobank-scale data. GigaScience.

[bib7] Codd V, Wang Q, Allara E, Musicha C, Kaptoge S, Stoma S, Jiang T, Hamby SE, Braund PS, Bountziouka V, Budgeon CA, Denniff M, Swinfield C, Papakonstantinou M, Sheth S, Nanus DE, Warner SC, Wang M, Khera AV, Eales J, Ouwehand WH, Thompson JR, Di Angelantonio E, Wood AM, Butterworth AS, Danesh JN, Nelson CP, Samani NJ (2021). Polygenic basis and biomedical consequences of telomere length variation. Nature Genetics.

[bib8] Colaprico A, Silva TC, Olsen C, Garofano L, Cava C, Garolini D, Sabedot TS, Malta TM, Pagnotta SM, Castiglioni I, Ceccarelli M, Bontempi G, Noushmehr H (2016). TCGAbiolinks: an R/bioconductor package for integrative analysis of TCGA data. Nucleic Acids Research.

[bib9] Das S, Forer L, Schönherr S, Sidore C, Locke AE, Kwong A, Vrieze SI, Chew EY, Levy S, McGue M, Schlessinger D, Stambolian D, Loh P-R, Iacono WG, Swaroop A, Scott LJ, Cucca F, Kronenberg F, Boehnke M, Abecasis GR, Fuchsberger C (2016). Next-generation genotype imputation service and methods. Nature Genetics.

[bib10] de Lange T (2009). How telomeres solve the end-protection problem. Science.

[bib11] Demanelis K, Jasmine F, Chen LS, Chernoff M, Tong L, Delgado D, Zhang C, Shinkle J, Sabarinathan M, Lin H, Ramirez E, Oliva M, Kim-Hellmuth S, Stranger BE, Lai TP, Aviv A, Ardlie KG, Aguet F, Ahsan H, Doherty JA, Kibriya MG, Pierce BL, GTEx Consortium (2020). Determinants of telomere length across human tissues. Science.

[bib12] Ellrott K, Bailey MH, Saksena G, Covington KR, Kandoth C, Stewart C, Hess J, Ma S, Chiotti KE, McLellan M, Sofia HJ, Hutter C, Getz G, Wheeler D, Ding L, MC3 Working Group, Cancer Genome Atlas Research Network (2018). Scalable open science approach for mutation calling of tumor exomes using multiple genomic pipelines. Cell Systems.

[bib13] Elsworth B, Lyon M, Alexander T, Liu Y, Matthews P, Hallett J, Bates P, Palmer T, Haberland V, Smith GD, Zheng J, Haycock P, Gaunt TR, Hemani G (2020). The MRC IEU OpenGWAS Data Infrastructure. bioRxiv.

[bib14] Foley CN, Staley JR, Breen PG, Sun BB, Kirk PDW, Burgess S, Howson JMM (2021). A fast and efficient colocalization algorithm for identifying shared genetic risk factors across multiple traits. Nature Communications.

[bib15] Gabriel AAG, Lipinski B (2021). GitHub.

[bib16] Gabriel AAG, Atkins JR, Penha RCC, Smith-Byrne K, Gaborieau V, Voegele C, Abedi-Ardekani B, Milojevic M, Olaso R, Meyer V, Boland A, Deleuze JF, Zaridze D, Mukeriya A, Swiatkowska B, Janout V, Schejbalová M, Mates D, Stojšić J, Ognjanovic M, Witte JS, Rashkin SR, Kachuri L, Hung RJ, Kar S, Brennan P, Sertier A-S, Ferrari A, Viari A, Johansson M, Amos CI, Foll M, McKay JD, the ILCCO consortium (2022). Genetic analysis of lung cancer and the germline impact on somatic mutation burden. JNCI.

[bib17] Giambartolomei C, Vukcevic D, Schadt EE, Franke L, Hingorani AD, Wallace C, Plagnol V (2014). Bayesian test for colocalisation between pairs of genetic association studies using summary statistics. PLOS Genetics.

[bib18] GTEx Consortium (2020). The gtex consortium atlas of genetic regulatory effects across human tissues. Science.

[bib19] Guo Y, Rall-Scharpf M, Bourdon JC, Wiesmüller L, Biber S (2021). P53 isoforms differentially impact on the POLι dependent DNA damage tolerance pathway. Cell Death & Disease.

[bib20] Hanahan D, Weinberg RA (2011). Hallmarks of cancer: the next generation. Cell.

[bib21] Harley CB, Futcher AB, Greider CW (1990). Telomeres shorten during ageing of human fibroblasts. Nature.

[bib22] Haycock PC, Burgess S, Nounu A, Zheng J, Okoli GN, Bowden J, Wade KH, Timpson NJ, Evans DM, Willeit P, Aviv A, Gaunt TR, Hemani G, Mangino M, Ellis HP, Kurian KM, Pooley KA, Eeles RA, Lee JE, Fang S, Chen WV, Law MH, Bowdler LM, Iles MM, Yang Q, Worrall BB, Markus HS, Hung RJ, Amos CI, Spurdle AB, Thompson DJ, O’Mara TA, Wolpin B, Amundadottir L, Stolzenberg-Solomon R, Trichopoulou A, Onland-Moret NC, Lund E, Duell EJ, Canzian F, Severi G, Overvad K, Gunter MJ, Tumino R, Svenson U, van Rij A, Baas AF, Bown MJ, Samani NJ, van t’Hof FNG, Tromp G, Jones GT, Kuivaniemi H, Elmore JR, Johansson M, Mckay J, Scelo G, Carreras-Torres R, Gaborieau V, Brennan P, Bracci PM, Neale RE, Olson SH, Gallinger S, Li D, Petersen GM, Risch HA, Klein AP, Han J, Abnet CC, Freedman ND, Taylor PR, Maris JM, Aben KK, Kiemeney LA, Vermeulen SH, Wiencke JK, Walsh KM, Wrensch M, Rice T, Turnbull C, Litchfield K, Paternoster L, Standl M, Abecasis GR, SanGiovanni JP, Li Y, Mijatovic V, Sapkota Y, Low S-K, Zondervan KT, Montgomery GW, Nyholt DR, van Heel DA, Hunt K, Arking DE, Ashar FN, Sotoodehnia N, Woo D, Rosand J, Comeau ME, Brown WM, Silverman EK, Hokanson JE, Cho MH, Hui J, Ferreira MA, Thompson PJ, Morrison AC, Felix JF, Smith NL, Christiano AM, Petukhova L, Betz RC, Fan X, Zhang X, Zhu C, Langefeld CD, Thompson SD, Wang F, Lin X, Schwartz DA, Fingerlin T, Rotter JI, Cotch MF, Jensen RA, Munz M, Dommisch H, Schaefer AS, Han F, Ollila HM, Hillary RP, Albagha O, Ralston SH, Zeng C, Zheng W, Shu X-O, Reis A, Uebe S, Hüffmeier U, Kawamura Y, Otowa T, Sasaki T, Hibberd ML, Davila S, Xie G, Siminovitch K, Bei J-X, Zeng Y-X, Försti A, Chen B, Landi S, Franke A, Fischer A, Ellinghaus D, Flores C, Noth I, Ma S-F, Foo JN, Liu J, Kim J-W, Cox DG, Delattre O, Mirabeau O, Skibola CF, Tang CS, Garcia-Barcelo M, Chang K-P, Su W-H, Chang Y-S, Martin NG, Gordon S, Wade TD, Lee C, Kubo M, Cha P-C, Nakamura Y, Levy D, Kimura M, Hwang S-J, Hunt S, Spector T, Soranzo N, Manichaikul AW, Barr RG, Kahali B, Speliotes E, Yerges-Armstrong LM, Cheng C-Y, Jonas JB, Wong TY, Fogh I, Lin K, Powell JF, Rice K, Relton CL, Martin RM, Davey Smith G, Telomeres Mendelian Randomization Collaboration (2017). Association between telomere length and risk of cancer and non-neoplastic diseases: a mendelian randomization study. JAMA Oncology.

[bib23] Hubert M, Branden KV (2003). Robust methods for partial least squares regression. Journal of Chemometrics.

[bib24] Hukku A, Pividori M, Luca F, Pique-Regi R, Im HK, Wen X (2021). Probabilistic colocalization of genetic variants from complex and molecular traits: promise and limitations. American Journal of Human Genetics.

[bib25] Kachuri L, Amos CI, McKay JD, Johansson M, Vineis P, Bueno-de-Mesquita HB, Boutron-Ruault M-C, Johansson M, Quirós JR, Sieri S, Travis RC, Weiderpass E, Le Marchand L, Henderson BE, Wilkens L, Goodman GE, Chen C, Doherty JA, Christiani DC, Wei Y, Su L, Tworoger S, Zhang X, Kraft P, Zaridze D, Field JK, Marcus MW, Davies MPA, Hyde R, Caporaso NE, Landi MT, Severi G, Giles GG, Liu G, McLaughlin JR, Li Y, Xiao X, Fehringer G, Zong X, Denroche RE, Zuzarte PC, McPherson JD, Brennan P, Hung RJ (2016). Fine mapping of chromosome 5p15.33 based on a targeted deep sequencing and high density genotyping identifies novel lung cancer susceptibility loci. Carcinogenesis.

[bib26] Kachuri L, Saarela O, Bojesen SE, Davey Smith G, Liu G, Landi MT, Caporaso NE, Christiani DC, Johansson M, Panico S, Overvad K, Trichopoulou A, Vineis P, Scelo G, Zaridze D, Wu X, Albanes D, Diergaarde B, Lagiou P, Macfarlane GJ, Aldrich MC, Tardón A, Rennert G, Olshan AF, Weissler MC, Chen C, Goodman GE, Doherty JA, Ness AR, Bickeböller H, Wichmann H-E, Risch A, Field JK, Teare MD, Kiemeney LA, van der Heijden EHFM, Carroll JC, Haugen A, Zienolddiny S, Skaug V, Wünsch-Filho V, Tajara EH, Ayoub Moysés R, Daumas Nunes F, Lam S, Eluf-Neto J, Lacko M, Peters WHM, Le Marchand L, Duell EJ, Andrew AS, Franceschi S, Schabath MB, Manjer J, Arnold S, Lazarus P, Mukeriya A, Swiatkowska B, Janout V, Holcatova I, Stojsic J, Mates D, Lissowska J, Boccia S, Lesseur C, Zong X, McKay JD, Brennan P, Amos CI, Hung RJ (2019). Mendelian randomization and mediation analysis of leukocyte telomere length and risk of lung and head and neck cancers. International Journal of Epidemiology.

[bib27] Kachuri L, Johansson M, Rashkin SR, Graff RE, Bossé Y, Manem V, Caporaso NE, Landi MT, Christiani DC, Vineis P, Liu G, Scelo G, Zaridze D, Shete SS, Albanes D, Aldrich MC, Tardón A, Rennert G, Chen C, Goodman GE, Doherty JA, Bickeböller H, Field JK, Davies MP, Dawn Teare M, Kiemeney LA, Bojesen SE, Haugen A, Zienolddiny S, Lam S, Le Marchand L, Cheng I, Schabath MB, Duell EJ, Andrew AS, Manjer J, Lazarus P, Arnold S, McKay JD, Emami NC, Warkentin MT, Brhane Y, Obeidat M, Martin RM, Relton C, Davey Smith G, Haycock PC, Amos CI, Brennan P, Witte JS, Hung RJ (2020). Immune-mediated genetic pathways resulting in pulmonary function impairment increase lung cancer susceptibility. Nature Communications.

[bib28] Knijnenburg TA, Wang L, Zimmermann MT, Chambwe N, Gao GF, Cherniack AD, Fan H, Shen H, Way GP, Greene CS, Liu Y, Akbani R, Feng B, Donehower LA, Miller C, Shen Y, Karimi M, Chen H, Kim P, Jia P, Shinbrot E, Zhang S, Liu J, Hu H, Bailey MH, Yau C, Wolf D, Zhao Z, Weinstein JN, Li L, Ding L, Mills GB, Laird PW, Wheeler DA, Shmulevich I, Monnat RJ, Xiao Y, Wang C, Cancer Genome Atlas Research Network (2018). Genomic and molecular landscape of DNA damage repair deficiency across the cancer genome atlas. Cell Reports.

[bib29] Landi MT, Chatterjee N, Yu K, Goldin LR, Goldstein AM, Rotunno M, Mirabello L, Jacobs K, Wheeler W, Yeager M, Bergen AW, Li Q, Consonni D, Pesatori AC, Wacholder S, Thun M, Diver R, Oken M, Virtamo J, Albanes D, Wang Z, Burdette L, Doheny KF, Pugh EW, Laurie C, Brennan P, Hung R, Gaborieau V, McKay JD, Lathrop M, McLaughlin J, Wang Y, Tsao M-S, Spitz MR, Wang Y, Krokan H, Vatten L, Skorpen F, Arnesen E, Benhamou S, Bouchard C, Metspalu A, Metsapalu A, Vooder T, Nelis M, Välk K, Field JK, Chen C, Goodman G, Sulem P, Thorleifsson G, Rafnar T, Eisen T, Sauter W, Rosenberger A, Bickeböller H, Risch A, Chang-Claude J, Wichmann HE, Stefansson K, Houlston R, Amos CI, Fraumeni JF, Savage SA, Bertazzi PA, Tucker MA, Chanock S, Caporaso NE (2009). A genome-wide association study of lung cancer identifies A region of chromosome 5p15 associated with risk for adenocarcinoma. American Journal of Human Genetics.

[bib30] Liu M, Jiang Y, Wedow R, Li Y, Brazel DM, Chen F, Datta G, Davila-Velderrain J, McGuire D, Tian C, Zhan X, Choquet H, Docherty AR, Faul JD, Foerster JR, Fritsche LG, Gabrielsen ME, Gordon SD, Haessler J, Hottenga J-J, Huang H, Jang S-K, Jansen PR, Ling Y, Mägi R, Matoba N, McMahon G, Mulas A, Orrù V, Palviainen T, Pandit A, Reginsson GW, Skogholt AH, Smith JA, Taylor AE, Turman C, Willemsen G, Young H, Young KA, Zajac GJM, Zhao W, Zhou W, Bjornsdottir G, Boardman JD, Boehnke M, Boomsma DI, Chen C, Cucca F, Davies GE, Eaton CB, Ehringer MA, Esko T, Fiorillo E, Gillespie NA, Gudbjartsson DF, Haller T, Harris KM, Heath AC, Hewitt JK, Hickie IB, Hokanson JE, Hopfer CJ, Hunter DJ, Iacono WG, Johnson EO, Kamatani Y, Kardia SLR, Keller MC, Kellis M, Kooperberg C, Kraft P, Krauter KS, Laakso M, Lind PA, Loukola A, Lutz SM, Madden PAF, Martin NG, McGue M, McQueen MB, Medland SE, Metspalu A, Mohlke KL, Nielsen JB, Okada Y, Peters U, Polderman TJC, Posthuma D, Reiner AP, Rice JP, Rimm E, Rose RJ, Runarsdottir V, Stallings MC, Stančáková A, Stefansson H, Thai KK, Tindle HA, Tyrfingsson T, Wall TL, Weir DR, Weisner C, Whitfield JB, Winsvold BS, Yin J, Zuccolo L, Bierut LJ, Hveem K, Lee JJ, Munafò MR, Saccone NL, Willer CJ, Cornelis MC, David SP, Hinds DA, Jorgenson E, Kaprio J, Stitzel JA, Stefansson K, Thorgeirsson TE, Abecasis G, Liu DJ, Vrieze S, 23andMe Research Team, HUNT All-In Psychiatry (2019). Association studies of up to 1.2 million individuals yield new insights into the genetic etiology of tobacco and alcohol use. Nature Genetics.

[bib31] Lopes K, Snijders GJL, Humphrey J, Allan A, Sneeboer MAM, Navarro E, Schilder BM, Vialle RA, Parks M, Missall R, van Zuiden W, Gigase FAJ, Kübler R, van Berlekom AB, Hicks EM, Bӧttcher C, Priller J, Kahn RS, de Witte LD, Raj T (2022). Genetic analysis of the human microglial transcriptome across brain regions, aging and disease pathologies. Nature Genetics.

[bib32] McIntyre J (2020). Polymerase iota - an odd sibling among Y family polymerases. DNA Repair.

[bib33] McKay JD, Hung RJ, Gaborieau V, Boffetta P, Chabrier A, Byrnes G, Zaridze D, Mukeria A, Szeszenia-Dabrowska N, Lissowska J, Rudnai P, Fabianova E, Mates D, Bencko V, Foretova L, Janout V, McLaughlin J, Shepherd F, Montpetit A, Narod S, Krokan HE, Skorpen F, Elvestad MB, Vatten L, Njølstad I, Axelsson T, Chen C, Goodman G, Barnett M, Loomis MM, Lubiñski J, Matyjasik J, Lener M, Oszutowska D, Field J, Liloglou T, Xinarianos G, Cassidy A, Vineis P, Clavel-Chapelon F, Palli D, Tumino R, Krogh V, Panico S, González CA, Ramón Quirós J, Martínez C, Navarro C, Ardanaz E, Larrañaga N, Kham KT, Key T, Bueno-de-Mesquita HB, Peeters PH, Trichopoulou A, Linseisen J, Boeing H, Hallmans G, Overvad K, Tjønneland A, Kumle M, Riboli E, Zelenika D, Boland A, Delepine M, Foglio M, Lechner D, Matsuda F, Blanche H, Gut I, Heath S, Lathrop M, Brennan P, EPIC Study (2008). Lung cancer susceptibility locus at 5p15.33. Nature Genetics.

[bib34] McKay JD, Hung RJ, Han Y, Zong X, Carreras-Torres R, Christiani DC, Caporaso NE, Johansson M, Xiao X, Li Y, Byun J, Dunning A, Pooley KA, Qian DC, Ji X, Liu G, Timofeeva MN, Bojesen SE, Wu X, Le Marchand L, Albanes D, Bickeböller H, Aldrich MC, Bush WS, Tardon A, Rennert G, Teare MD, Field JK, Kiemeney LA, Lazarus P, Haugen A, Lam S, Schabath MB, Andrew AS, Shen H, Hong Y-C, Yuan J-M, Bertazzi PA, Pesatori AC, Ye Y, Diao N, Su L, Zhang R, Brhane Y, Leighl N, Johansen JS, Mellemgaard A, Saliba W, Haiman CA, Wilkens LR, Fernandez-Somoano A, Fernandez-Tardon G, van der Heijden HFM, Kim JH, Dai J, Hu Z, Davies MPA, Marcus MW, Brunnström H, Manjer J, Melander O, Muller DC, Overvad K, Trichopoulou A, Tumino R, Doherty JA, Barnett MP, Chen C, Goodman GE, Cox A, Taylor F, Woll P, Brüske I, Wichmann H-E, Manz J, Muley TR, Risch A, Rosenberger A, Grankvist K, Johansson M, Shepherd FA, Tsao M-S, Arnold SM, Haura EB, Bolca C, Holcatova I, Janout V, Kontic M, Lissowska J, Mukeria A, Ognjanovic S, Orlowski TM, Scelo G, Swiatkowska B, Zaridze D, Bakke P, Skaug V, Zienolddiny S, Duell EJ, Butler LM, Koh W-P, Gao Y-T, Houlston RS, McLaughlin J, Stevens VL, Joubert P, Lamontagne M, Nickle DC, Obeidat M, Timens W, Zhu B, Song L, Kachuri L, Artigas MS, Tobin MD, Wain LV, Rafnar T, Thorgeirsson TE, Reginsson GW, Stefansson K, Hancock DB, Bierut LJ, Spitz MR, Gaddis NC, Lutz SM, Gu F, Johnson EO, Kamal A, Pikielny C, Zhu D, Lindströem S, Jiang X, Tyndale RF, Chenevix-Trench G, Beesley J, Bossé Y, Chanock S, Brennan P, Landi MT, Amos CI, SpiroMeta Consortium (2017). Large-scale association analysis identifies new lung cancer susceptibility loci and heterogeneity in genetic susceptibility across histological subtypes. Nature Genetics.

[bib35] Montpetit AJ, Alhareeri AA, Montpetit M, Starkweather AR, Elmore LW, Filler K, Mohanraj L, Burton CW, Menzies VS, Lyon DE, Jackson-Cook CK (2014). Telomere length: a review of methods for measurement. Nursing Research.

[bib36] Penha RCC (2023a). Software Heritage.

[bib37] Penha RCC (2023b). Software Heritage.

[bib38] Pulit SL, Stoneman C, Morris AP, Wood AR, Glastonbury CA, Tyrrell J, Yengo L, Ferreira T, Marouli E, Ji Y, Yang J, Jones S, Beaumont R, Croteau-Chonka DC, Winkler TW, Hattersley AT, Loos RJF, Hirschhorn JN, Visscher PM, Frayling TM, Yaghootkar H, Lindgren CM, GIANT Consortium (2019). Meta-analysis of genome-wide association studies for body fat distribution in 694 649 individuals of european ancestry. Human Molecular Genetics.

[bib39] Rafnar T, Sulem P, Stacey SN, Geller F, Gudmundsson J, Sigurdsson A, Jakobsdottir M, Helgadottir H, Thorlacius S, Aben KKH, Blöndal T, Thorgeirsson TE, Thorleifsson G, Kristjansson K, Thorisdottir K, Ragnarsson R, Sigurgeirsson B, Skuladottir H, Gudbjartsson T, Isaksson HJ, Einarsson GV, Benediktsdottir KR, Agnarsson BA, Olafsson K, Salvarsdottir A, Bjarnason H, Asgeirsdottir M, Kristinsson KT, Matthiasdottir S, Sveinsdottir SG, Polidoro S, Höiom V, Botella-Estrada R, Hemminki K, Rudnai P, Bishop DT, Campagna M, Kellen E, Zeegers MP, de Verdier P, Ferrer A, Isla D, Vidal MJ, Andres R, Saez B, Juberias P, Banzo J, Navarrete S, Tres A, Kan D, Lindblom A, Gurzau E, Koppova K, de Vegt F, Schalken JA, van der Heijden HFM, Smit HJ, Termeer RA, Oosterwijk E, van Hooij O, Nagore E, Porru S, Steineck G, Hansson J, Buntinx F, Catalona WJ, Matullo G, Vineis P, Kiltie AE, Mayordomo JI, Kumar R, Kiemeney LA, Frigge ML, Jonsson T, Saemundsson H, Barkardottir RB, Jonsson E, Jonsson S, Olafsson JH, Gulcher JR, Masson G, Gudbjartsson DF, Kong A, Thorsteinsdottir U, Stefansson K (2009). Sequence variants at the TERT-CLPTM1L locus associate with many cancer types. Nature Genetics.

[bib40] Risso D, Schwartz K, Sherlock G, Dudoit S (2011). Gc-Content normalization for RNA-Seq data. BMC Bioinformatics.

[bib41] Sainz de Aja J, Dost AFM, Kim CF (2021). Alveolar progenitor cells and the origin of lung cancer. Journal of Internal Medicine.

[bib42] Sanchez-Espiridion B, Chen M, Chang JY, Lu C, Chang DW, Roth JA, Wu X, Gu J (2014). Telomere length in peripheral blood leukocytes and lung cancer risk: a large case-control study in caucasians. Cancer Research.

[bib43] Sanderson E, Davey Smith G, Windmeijer F, Bowden J (2019). An examination of multivariable Mendelian randomization in the single-sample and two-sample summary data settings. International Journal of Epidemiology.

[bib44] Sanderson E, Glymour MM, Holmes MV, Kang H, Morrison J, Munafò MR, Palmer T, Schooling CM, Wallace C, Zhao Q, Davey Smith G (2022). Mendelian randomization. Nature Reviews Methods Primers.

[bib45] Saunders GRB, Wang X, Chen F, Jang S-K, Liu M, Wang C, Gao S, Jiang Y, Khunsriraksakul C, Otto JM, Addison C, Akiyama M, Albert CM, Aliev F, Alonso A, Arnett DK, Ashley-Koch AE, Ashrani AA, Barnes KC, Barr RG, Bartz TM, Becker DM, Bielak LF, Benjamin EJ, Bis JC, Bjornsdottir G, Blangero J, Bleecker ER, Boardman JD, Boerwinkle E, Boomsma DI, Boorgula MP, Bowden DW, Brody JA, Cade BE, Chasman DI, Chavan S, Chen Y-DI, Chen Z, Cheng I, Cho MH, Choquet H, Cole JW, Cornelis MC, Cucca F, Curran JE, de Andrade M, Dick DM, Docherty AR, Duggirala R, Eaton CB, Ehringer MA, Esko T, Faul JD, Fernandes Silva L, Fiorillo E, Fornage M, Freedman BI, Gabrielsen ME, Garrett ME, Gharib SA, Gieger C, Gillespie N, Glahn DC, Gordon SD, Gu CC, Gu D, Gudbjartsson DF, Guo X, Haessler J, Hall ME, Haller T, Harris KM, He J, Herd P, Hewitt JK, Hickie I, Hidalgo B, Hokanson JE, Hopfer C, Hottenga J, Hou L, Huang H, Hung Y-J, Hunter DJ, Hveem K, Hwang S-J, Hwu C-M, Iacono W, Irvin MR, Jee YH, Johnson EO, Joo YY, Jorgenson E, Justice AE, Kamatani Y, Kaplan RC, Kaprio J, Kardia SLR, Keller MC, Kelly TN, Kooperberg C, Korhonen T, Kraft P, Krauter K, Kuusisto J, Laakso M, Lasky-Su J, Lee W-J, Lee JJ, Levy D, Li L, Li K, Li Y, Lin K, Lind PA, Liu C, Lloyd-Jones DM, Lutz SM, Ma J, Mägi R, Manichaikul A, Martin NG, Mathur R, Matoba N, McArdle PF, McGue M, McQueen MB, Medland SE, Metspalu A, Meyers DA, Millwood IY, Mitchell BD, Mohlke KL, Moll M, Montasser ME, Morrison AC, Mulas A, Nielsen JB, North KE, Oelsner EC, Okada Y, Orrù V, Palmer ND, Palviainen T, Pandit A, Park SL, Peters U, Peters A, Peyser PA, Polderman TJC, Rafaels N, Redline S, Reed RM, Reiner AP, Rice JP, Rich SS, Richmond NE, Roan C, Rotter JI, Rueschman MN, Runarsdottir V, Saccone NL, Schwartz DA, Shadyab AH, Shi J, Shringarpure SS, Sicinski K, Skogholt AH, Smith JA, Smith NL, Sotoodehnia N, Stallings MC, Stefansson H, Stefansson K, Stitzel JA, Sun X, Syed M, Tal-Singer R, Taylor AE, Taylor KD, Telen MJ, Thai KK, Tiwari H, Turman C, Tyrfingsson T, Wall TL, Walters RG, Weir DR, Weiss ST, White WB, Whitfield JB, Wiggins KL, Willemsen G, Willer CJ, Winsvold BS, Xu H, Yanek LR, Yin J, Young KL, Young KA, Yu B, Zhao W, Zhou W, Zöllner S, Zuccolo L, Batini C, Bergen AW, Bierut LJ, David SP, Gagliano Taliun SA, Hancock DB, Jiang B, Munafò MR, Thorgeirsson TE, Liu DJ, Vrieze S, 23andMe Research Team, Biobank Japan Project (2022). Genetic diversity fuels gene discovery for tobacco and alcohol use. Nature.

[bib46] Subramanian A, Tamayo P, Mootha VK, Mukherjee S, Ebert BL, Gillette MA, Paulovich A, Pomeroy SL, Golub TR, Lander ES, Mesirov JP (2005). Gene set enrichment analysis: a knowledge-based approach for interpreting genome-wide expression profiles. PNAS.

[bib47] Thorsson V, Gibbs DL, Brown SD, Wolf D, Bortone DS, Ou Yang T-H, Porta-Pardo E, Gao GF, Plaisier CL, Eddy JA, Ziv E, Culhane AC, Paull EO, Sivakumar IKA, Gentles AJ, Malhotra R, Farshidfar F, Colaprico A, Parker JS, Mose LE, Vo NS, Liu J, Liu Y, Rader J, Dhankani V, Reynolds SM, Bowlby R, Califano A, Cherniack AD, Anastassiou D, Bedognetti D, Mokrab Y, Newman AM, Rao A, Chen K, Krasnitz A, Hu H, Malta TM, Noushmehr H, Pedamallu CS, Bullman S, Ojesina AI, Lamb A, Zhou W, Shen H, Choueiri TK, Weinstein JN, Guinney J, Saltz J, Holt RA, Rabkin CS, Lazar AJ, Serody JS, Demicco EG, Disis ML, Vincent BG, Shmulevich I, Cancer Genome Atlas Research Network (2018). The immune landscape of cancer. Immunity.

[bib48] Wallace C (2020). Eliciting priors and relaxing the single causal variant assumption in colocalisation analyses. PLOS Genetics.

[bib49] Wallace C (2021). A more accurate method for colocalisation analysis allowing for multiple causal variants. PLOS Genetics.

[bib50] Wang Y, Broderick P, Webb E, Wu X, Vijayakrishnan J, Matakidou A, Qureshi M, Dong Q, Gu X, Chen WV, Spitz MR, Eisen T, Amos CI, Houlston RS (2008). Common 5p15.33 and 6p21.33 variants influence lung cancer risk. Nature Genetics.

[bib51] Watson JD (1972). Origin of concatemeric T7 DNA. Nature.

[bib52] Yavorska OO, Burgess S (2017). MendelianRandomization: an R package for performing Mendelian randomization analyses using summarized data. International Journal of Epidemiology.

[bib53] Zhang C, Doherty JA, Burgess S, Hung RJ, Lindström S, Kraft P, Gong J, Amos CI, Sellers TA, Monteiro ANA, Chenevix-Trench G, Bickeböller H, Risch A, Brennan P, Mckay JD, Houlston RS, Landi MT, Timofeeva MN, Wang Y, Heinrich J, Kote-Jarai Z, Eeles RA, Muir K, Wiklund F, Grönberg H, Berndt SI, Chanock SJ, Schumacher F, Haiman CA, Henderson BE, Amin Al Olama A, Andrulis IL, Hopper JL, Chang-Claude J, John EM, Malone KE, Gammon MD, Ursin G, Whittemore AS, Hunter DJ, Gruber SB, Knight JA, Hou L, Le Marchand L, Newcomb PA, Hudson TJ, Chan AT, Li L, Woods MO, Ahsan H, Pierce BL (2015). Genetic determinants of telomere length and risk of common cancers: a Mendelian randomization study. Human Molecular Genetics.

[bib54] Zhang X, Zhao Q, Zhu W, Liu T, Xie SH, Zhong LX, Cai YY, Li XN, Liang M, Chen W, Hu QS, Zhang B (2017). The association of telomere length in peripheral blood cells with cancer risk: a systematic review and meta-analysis of prospective studies. Cancer Epidemiology, Biomarkers & Prevention.

